# Determinants of selection in yeast evolved by genome shuffling

**DOI:** 10.1186/s13068-018-1283-9

**Published:** 2018-10-16

**Authors:** Damien Biot-Pelletier, Dominic Pinel, Kane Larue, Vincent J. J. Martin

**Affiliations:** 1 Department of Biology, Centre for Structural and Functional Genomics, Centre for Applied Synthetic Biology, Concordia University, 7141 Sherbrooke Street West, Montreal, QC H4B 1R6 Canada; 2 Present Address: Lallemand Inc., Montréal, QC H4P 2R2 Canada; 3 Present Address: Amyris Inc, Emeryville, CA 94608 USA; 4 Present Address: Charles River Laboratories, Senneville, QC H9X 3R3 Canada

**Keywords:** Evolutionary engineering, Genome shuffling, Evolution, Lignocellulosic hydrolysate tolerance, Stress tolerance in yeast

## Abstract

**Background:**

Genome shuffling (GS) is a widely adopted methodology for the evolutionary engineering of desirable traits in industrially relevant microorganisms. We have previously used genome shuffling to generate a strain of *Saccharomyces cerevisiae* that is tolerant to the growth inhibitors found in a lignocellulosic hydrolysate. In this study, we expand on previous work by performing a population-wide genomic survey of our genome shuffling experiment and dissecting the molecular determinants of the evolved phenotype.

**Results:**

Whole population whole-genome sequencing was used to survey mutations selected during the experiment and extract allele frequency time series. Using growth curve assays on single point mutants and backcrossed derivatives, we explored the genetic architecture of the selected phenotype and detected examples of epistasis. Our results reveal cohorts of strongly correlated mutations, suggesting prevalent genetic hitchhiking and the presence of pre-existing founder mutations. From the patterns of apparent selection and the results of direct phenotypic assays, our results identify key driver mutations and deleterious hitchhikers.

**Conclusions:**

We use these data to propose a model of inhibitor tolerance in our GS mutants. Our results also suggest a role for compensatory evolution and epistasis in our genome shuffling experiment and illustrate the impact of historical contingency on the outcomes of evolutionary engineering.

**Electronic supplementary material:**

The online version of this article (10.1186/s13068-018-1283-9) contains supplementary material, which is available to authorized users.

## Background

**Fig. 1:**
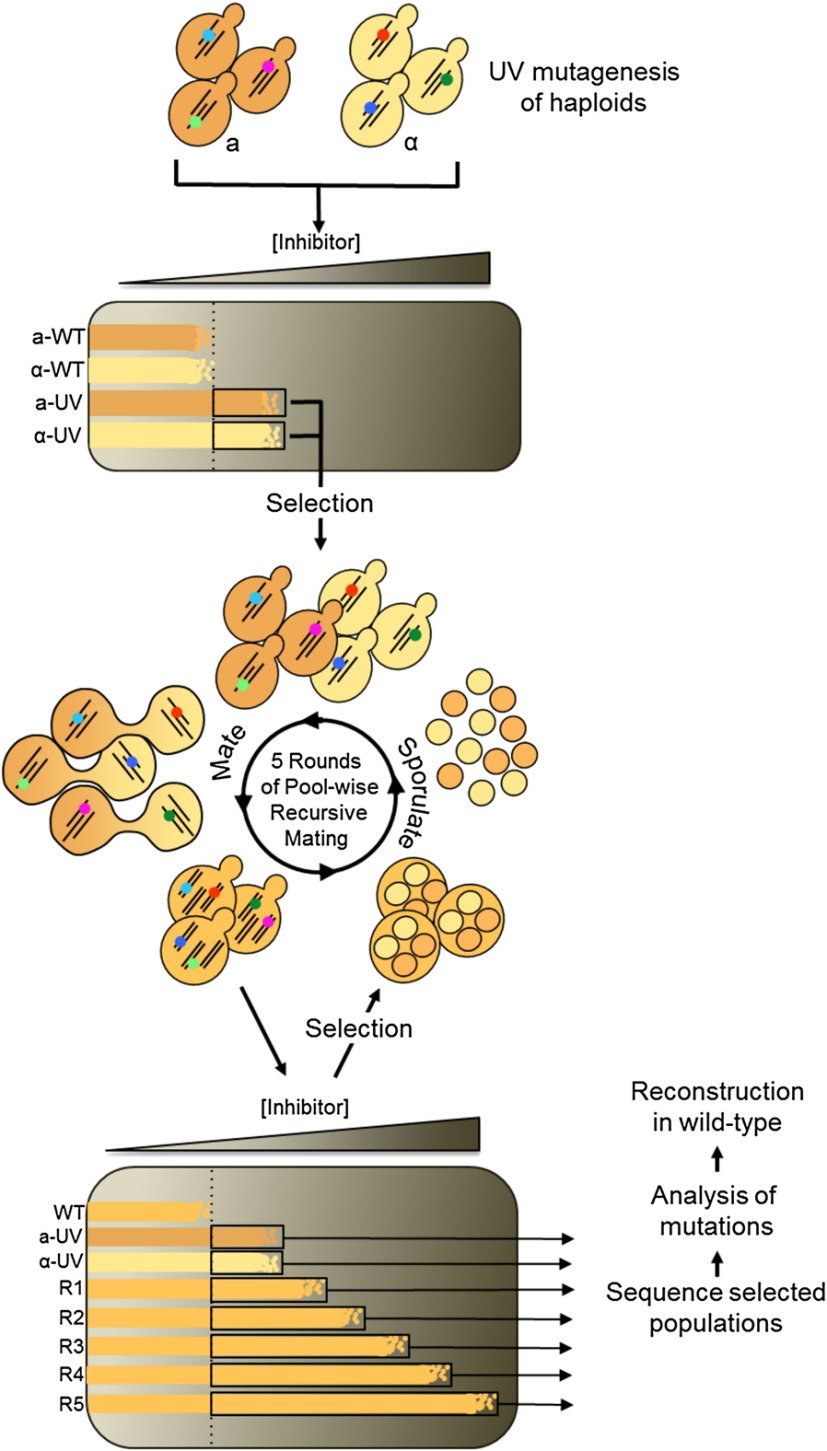
Outline of the genome shuffling experiment. Wild-type haploid cells of both mating types were UV irradiated to generate pools of haploid mutants. Mutants with tolerance to hydrolysate superior to their wild-type ancestors were selected using plates displaying a gradient of hydrolysate concentration. The pools of tolerant haploid mutants thus obtained were mated, generating diploids. Tolerant diploids were selected on gradient plates, and sporulated. Resulting haploids were mated at random, effecting the shuffling of mutations. Five cycles of diploid selection on gradient plates, sporulation and mating were performed. Genomic DNA from each round of shuffling and selection was submitted to population genome sequencing and mutation analysis

Genome shuffling (GS) is an evolutionary engineering method based on recursive recombination and selection in populations of mutants (Fig. 1). It aims to speed the rate of evolution of desired traits by exploiting sexual, parasexual or artificial recombination to promote purifying selection, positive epistasis, and the accumulation of beneficial mutations, while reducing clonal interference. It has been widely and successfully adopted for the evolutionary engineering of industrial traits in microbes [1]. GS and other evolutionary engineering methodologies are notably useful to enhance complex phenotypes for which a detailed molecular level understanding is lacking. Studies aimed at uncovering the genetic architecture of strains evolved by genome shuffling may thus contribute to an understanding of the genetic basis of complex and industrially relevant traits. Targeted approaches such as candidate gene sequencing [2] and qPCR [3, 4] have been used to uncover the genetic determinants of traits evolved by GS. System-level approaches, like array-comparative genome hybridization [5], RNAseq [5, 6], and whole genome sequencing [6–8], as well as proteomics methods [9–11] have also been used to investigate the complex genetic architecture of strains derived from GS experiments.

Massively parallel sequencing technologies have enabled monitoring of the appearance, frequency and fluctuation of mutant alleles in experimental evolution experiments [12]. A growing body of work on adaptive evolution takes advantage of evolve-and-resequence experiments to explore evolutionary dynamics under various sets of experimental constraints and environments [13]. Several evolutionary behaviors and dynamics predicted by evolutionary theory and their effects on adaptation have been illustrated in this way. Notable examples include clonal interference and genetic hitchhiking in asexual populations [14], the effect of sign epistasis on adaptive landscapes [15] and the mechanisms by which sexual recombination speeds adaptation [16]. Recently, whole population sequencing of evolutionary time points has been extended to the study of industrially relevant phenotypes, probing the dynamics and molecular processes affected during adaptation of *S. cerevisiae* to high ethanol stress [17]. This study identified specific mutations conferring increased tolerance to high ethanol and illustrated the diversity of evolutionary mechanisms involved in the adaptive response to complex stresses.

Most evolve-and-resequence studies have studied evolution from isogenic starting populations of asexually reproducing microbes [13]. These experimental setups, in which selection is applied on diversity that strictly derives from de novo mutations, have demonstrated pervasive clonal interference and widespread genetic hitchhiking [14, 18, 19]. Parallel evolution is observed in these experiments, despite high levels of molecular diversity [20–22]. More relevant to the context of genome shuffling, evolution of *S. cerevisiae* populations with sexual reproduction and high levels of initial diversity were shown to follow similarly deterministic paths while granting a marginal role for de novo mutations [23]. A similar methodology was used to compare the rate and dynamics of adaptation in the absence and presence of sexual recombination events, showing that sex accelerates the rate of adaptation by reducing clonal interference and enabling efficient purifying selection [16].

We have previously used GS to successfully evolve strains of *Saccharomyces cerevisiae* tolerant to spent sulfite liquor (SSL), a toxic lignocellulosic hydrolysate and byproduct of the acid bisulfite wood pulping process [24]. These mutants were characterized as highly tolerant to osmotic and oxidative stresses, organic acids and phenolic compounds. A strain, designated R57, with high inhibitor tolerance and ability to ferment hydrolysate sugars to ethanol was identified [6]. Whole-genome sequencing, RNAseq and whole population amplicon sequencing were used to probe the genetic architecture of R57. The strain differs from its parent by 21 single nucleotide changes affecting 17 genes [6].

This study aims to explore the evolutionary dynamics of genome shuffling by identifying the molecular and evolutionary determinants of selection in our experiment. To our knowledge, it is the first study to use genome sequencing of a GS population at several evolutionary time points. It completes our survey of selected mutations and retrieves allele frequency time series spanning our evolutionary engineering experiment. Inhibitor tolerance assays and genotyping by amplicon sequencing of meiotic segregants of R57 provided data for a multivariable linear model predicting the contribution of individual mutations to the hydrolysate tolerance phenotype. The phenotypic effects of single mutations reconstituted in wild type or deleted from the R57 background were also tested. Based on these results, we propose a descriptive model of the evolutionary dynamics of our GS experiment. We discuss the impact of historical contingency and compensatory evolution on the outcomes of GS, and demonstrate prevalent genetic hitchhiking. We also identify key genetic determinants of hydrolysate inhibitor tolerance.

## Results

### Pooled sequencing of evolving populations

Mutant populations with increased tolerance to hydrolysate inhibitors were generated by genome shuffling as described in Fig. 1 and previous publications [6, 24]. In short, two pools of random mutants were generated by UV mutagenesis of wild-type *MATa* and *MATα* haploids. Haploid mutants with tolerance above wild-type levels were selected on gradient plates, which consist in dishes of agar medium displaying increasing concentration of hydrolysate from one end to the other. *MATa* and *MATα* mutants were mated to generate diploids carrying random combinations of mutations. These diploids were selected on gradient plates to enrich for individuals with superior hydrolysate tolerance. After mating, diploids were sporulated, digesting and sonicating to disrupt asci and eliminate non-sporulated vegetative cells. Resulting haploids were mated to effect genome shuffling. In total, the mutant pool was submitted to 5 rounds of recursive mating, selection, and sporulation, generating populations with increasing tolerance to hydrolysate inhibitors. Strain R57, which displays high tolerance to hydrolysate inhibitors, was isolated from the fifth and final round of genome shuffling. Its genome differs from the CEN.PK113-7D reference sequence [25] by the 21 single nucleotide substitutions listed in Additional file 1: Table S2.

To gain insight into the genetic landscape of our populations evolved by genome shuffling, we investigated the metagenome of seven populations from six time points (Fig. 1). For sequencing, we selected time points covering the entire length of the experiment, including both populations of selected UV haploids and shuffled mutants from each of the five rounds of genome shuffling (R1–R5). Each population was re-sequenced, generating upwards of 300 million reads, for an average of 40 billion nucleotides per sample with a mean base quality score of 35.07 (Additional file 1: Table S1). The 100 nucleotide reads were aligned to the CEN.PK113-7D reference genome [25], which is one of the parental strains used in the experiment. Mean depth of coverage oscillated between 712× and 1551×, for an average of 1091×, enabling the detection of SNPs represented in < 1% of the population (Additional file 1: Table S1).

**Fig. 2:**
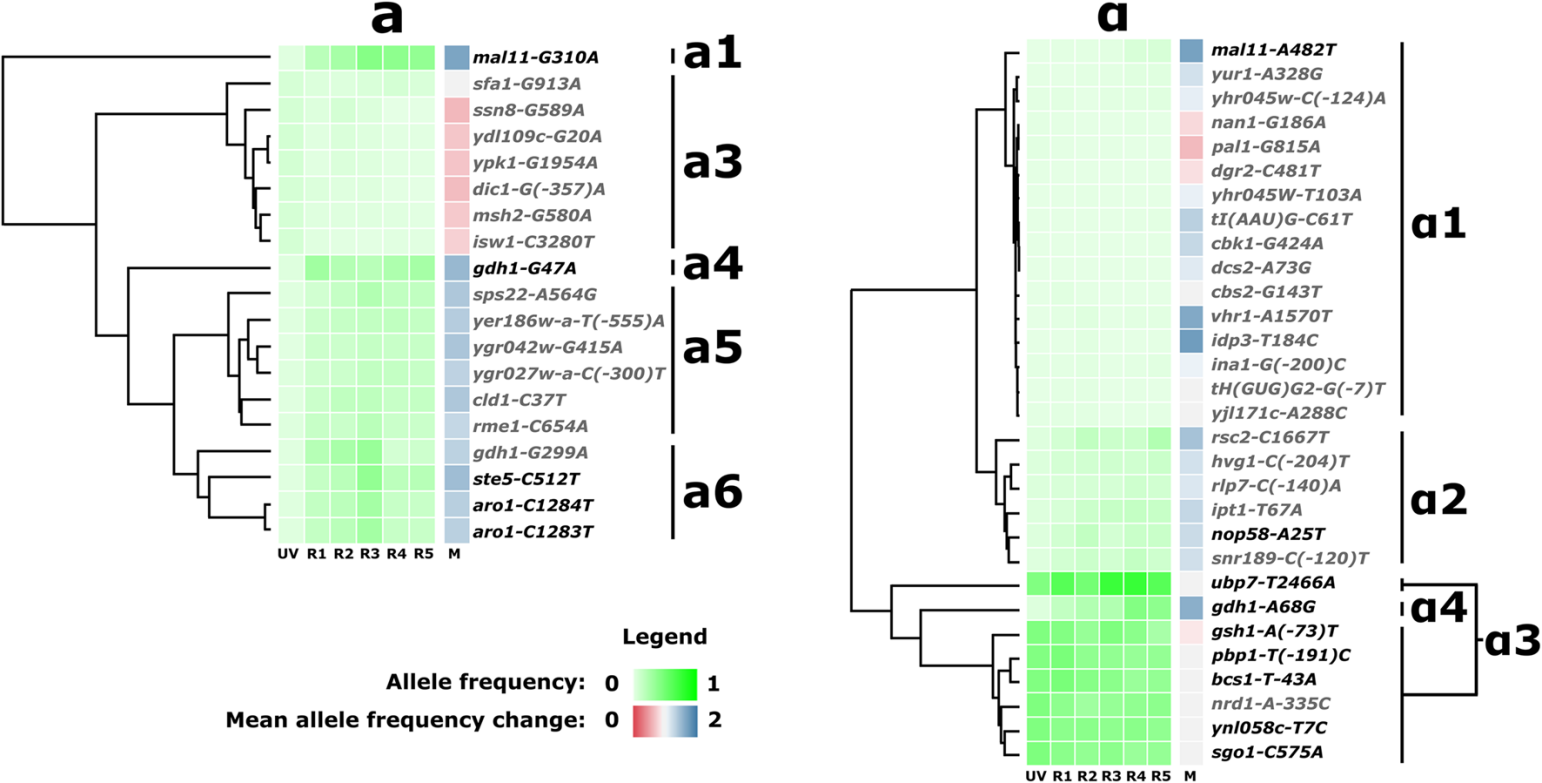
Evolutionary trajectories of the most prominent clusters of mutations. Mutations arose either in the *MATa* (left) or *MATα* (right) pools. On the vertical axis are the names of the mutations, giving the closest gene, coordinates relative to that gene, and the nature of the nucleotide substitution. On the horizontal axis are each of the six evolutionary timepoints (UV, R1, R2, R3, R4, R5), and the mean allele frequency change (*M*). Frequency of the mutant alleles is represented by shades of green. Mean allele frequency changes are represented in shades of red (*M* < 1, declining frequency) to blue (*M* > 1, increasing frequency). Hierarchical clustering of individual evolutionary trajectories is represented by dendrograms on the left. Mutations were assigned to cohorts of mutations (a1-5, α1-3) on the basis of this clustering. Mutations present in highly tolerant mutant R57 are highlighted in bold. The large and low frequency cohort a2 is omitted from the figure for clarity and brevity. See Additional file 3: Figure S1 for full dataset

A base error model was used for calling SNPs, distinguishing genuine mutations from sequencing errors. Filtering and manual examination resulted in a list of 188 SNPs (Tab “All_mutations” in Additional file 2). A further reduced list was prepared by excluding mutations that either were synonymous or escaped detection in at least one of the six time points (Tab “Non_synonymous_non_zero” in Additional file 2). We detected the majority of SNPs previously identified in R57 [6] most of which are found at medium to high frequency in the re-sequenced populations (Fig. 2). Previous sequencing of strain R57 found mutations in genes *TOF2*, *DOP1* and *FIT3*, but they could not be detected by population sequencing, implying that their frequencies fall below our detection threshold.

### Correlated evolutionary trajectories suggest genetic hitchhiking

All the 105 SNPs have their origin in either the *MATa* (33 SNPs) or *MATα* (72 SNPs) parental strains (Fig. 2 and Additional file 3: Figure S1). Visual examination of the allele frequencies suggested that SNPs could be further clustered in cohorts with strongly correlated evolutionary trajectories. For example, mutations *aro1*-*CC1283*-*4TT* and *ste5*-*C512T*, both on chromosome IV, display similar frequencies at all time points and originate in the *MATa* population. This observation suggested the existence of subgroups of SNPs of common origin hitchhiking on a few driver mutations. To test this hypothesis and identify cohorts of SNPs potentially linked by origin, we performed hierarchical clustering on the evolutionary trajectories of all SNPs (Fig. 2 and in Additional file 3: Figure S1). Nine cohorts of mutations were deduced from the resulting dendrograms. The majority of mutations are found at very low frequency (≤ 0.02) in all sampled time points, with varying levels of apparent selection. Those mutations are assigned to cohorts α1 and a2.

Three mutations show unique trajectories. Mutation *mal11*-*G310A* stands out as displaying the strongest apparent selection, with a mean allele frequency change of 1.69. Similarly, the *gdh1*-*G47A* mutation, with a mean allele frequency change of 1.53, is not clustered with other mutations. The *gdh1*-*A68G* mutation, displaying one of the strongest apparent selections (*M* = 1.60), is placed with the α3 cluster by the algorithm, but its trajectory is markedly different from other mutations in that cohort. It has a much lower initial frequency and displays stronger positive selection. We, therefore, also assigned *gdh1*-*A68G* to its own cohort (α4).

The most frequent *MATa*-derived cohorts are a5 and a6. In both cohorts, mutations start at a frequency of approximately 0.03 and increase steadily to reach maximum frequency after 3 cycles of mating and selection (R3) followed by a decline in subsequent cycles (R4 and R5). With similar trajectories but varying allele frequencies, it is not clear whether SNPs clustered in a5 and a6 belong to two independent cohorts or a single group of hitchhikers.

Cohort α3 consists of seven SNPs remarkable for their high and virtually identical initial frequency. SNPs from this group display a frequency of ~ 0.88 in the *MATα* population, indicating that they nearly swept the population at early selection steps. This group benefits from the founder effect, remaining highly represented until the end of the experiment. The general pattern followed by all but one mutation (*ubp7*-*T2466A*) suggests an absence of selection, or a slow decline in frequency (*M* = 0.903–0.970). The mutation in gene *UBP7* diverges from the rest of the group, on average increasing in frequency. Large increases in frequency in the early mating and selection cycles (0.49–0.74 between R2 and R3) are followed by a decline, amounting to a modest measure of apparent selection for *ubp7*-*T2466A (M* = 1.070).

Five SNPs were detected in all reads of their original mutant population; *srb8*-*C3787G* and *art5*-*G454T* in the *MATa* and *mtm1*-*A943T*, *avl9*-*C1806G* and *sro77*-*G(*-*160)T* in the *MATα*. The frequency of these mutations oscillates around 0.50 in all 5 genome shuffled populations, indicating relative neutrality with respect to the selected phenotype (Additional file 2). These two observations suggest that they spontaneously arose in the parental clones before UV mutagenesis.

The signature of genetic hitchhiking observed in our data indicates that a large proportion of the SNPs identified by sequencing arose together in a few founding individuals. From the list of mutations detected by population sequencing, a restricted subset of mutations is thus expected to contribute to the phenotype of interest.

### Certain genes are mutation hotspots

We observed several independent occurrences of distinct point mutations mapping to the same genes. A systematic survey revealed eight genes to which more than one mutation could be mapped (Additional file 4: Figure S2). Four strongly correlated mutations in genes *STE5* and *ARO1* were excluded because their correlated evolutionary trajectories suggested that they resulted from the same mutation event. Among the 25 remaining SNPs, 9 escape detection in at least one of the sampled time points (Tab “All_mutations” in Additional file 2). Most hotspot mutations displayed low frequency, with the exception of four SNPs, one of which mapped to *MAL11* and the three others to *GDH1*. Mutations mapping to these two genes, as well as those mapping to *NRG1*, display a pattern of strongly positive apparent selection (Additional file 4: Figure S2). Remaining hotspots, mapping to genes *VHR1*, *SSN2*, *YHR045W*, *UBP1* and *COS111* display either negative or nearly neutral selection.

**Fig. 3:**
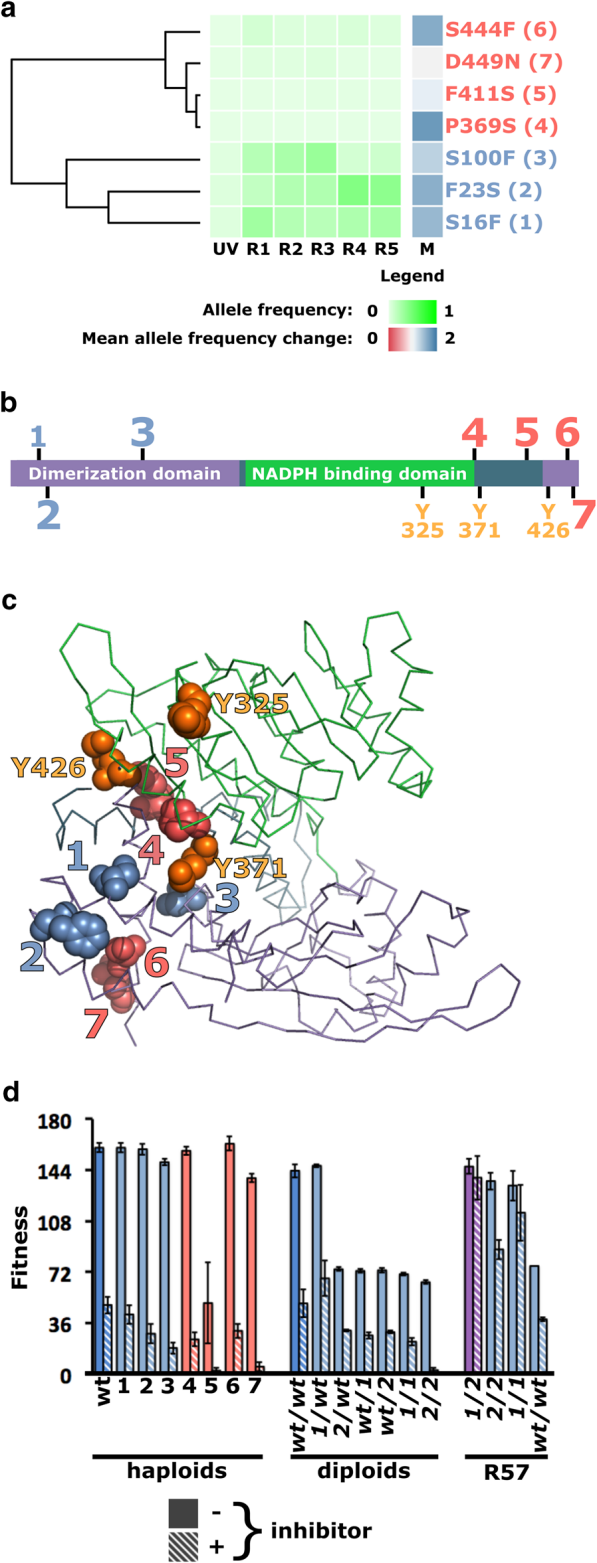
Mutations in *GDH1* played a critical role in the evolution of SSL tolerance. **a** Seven independent mutations result in amino acid substitutions in the Gdh1 protein. Their evolutionary trajectories cluster into a high (blue) and low (red) frequency group. **b** The high-frequency group maps at the N-terminus of Gdh1p, while the low frequency cohort affects residues clustered at the C-terminus. **c** Mapping of the seven substitutions onto a homology model of Gdh1p reveals that they all cluster in or near the hinge connecting the two structural domains of the protein, an area rich with known ubiquitination sites (displayed in orange). **d** Introduction of the *gdh1* mutations into haploid and diploid wild-type backgrounds leads to a loss of fitness. Removal of mutant *gdh1* alleles from the SSL-tolerant R57 background is also associated with a loss of tolerance. Error bars represent ± 1 standard deviation

The most remarkable hotspot maps seven non-synonymous mutations to *GDH1*. A strong positive selection was observed for most of these mutations and three of them rapidly rose to prominence in the course of evolution (Fig. 3a). Three mutations cluster at the N-terminus of Gdh1p and four at the C-terminus (Fig. 3b). Multiple sequence alignment of Gdh1p homologs did not suggest a high level of sequence conservation for the affected residues (data not shown), but mapping of Gdh1p substitutions on a homology model (see Additional file 5) showed that both N- and C-terminal substitutions are grouped near the hinge region separating the two structural domains of the protein (Fig. 3c). Together, these observations convincingly argue for the strong selection of *gdh1* alleles by our genome shuffling experiment.

To test the effect of *gdh1* mutations on the phenotype, the seven-point mutants were reconstructed in a wild-type parental haploid background. Under non-selective conditions, with the exception of the crippling D411S mutation, all haploid *gdh1* mutants grow as well as their wild-type parent but showed decreased fitness upon expose to the inhibitory substrate (Fig. 3d). Reversion of the S16F or F23S mutations found in strain R57 did not alter growth in permissive conditions, but resulted in loss of fitness in a wild-type homozygous background (Fig. 3d). In R57, the *GDH1/GDH1* genotype is associated with a growth defect both in the presence and absence of inhibitors. Mutations in gene *GDH1* are important for the inhibitor tolerance phenotype of R57, with reduced tolerance most obvious when S16F is reverted either alone or in combination with F23S. Together, these observations suggest reciprocal sign epistasis between *gdh1* alleles and the wild-type and mutant backgrounds.

### Effect of individual mutations on inhibitor tolerance phenotype

To estimate the contribution of individual mutations on the selected phenotype, we backcrossed random haploid segregants of R57 with the wild-type *MATα* parent strain and the fitness of 86 second-generation derivatives was assayed under permissive and inhibitory conditions. The result revealed a continuous distribution in their level of tolerance, with some mutants displaying a phenotype superior to the R57 parent (Additional file 6: Figure S3). A Kolmogorov–Smirnov test (*α* = 0.05) suggests a normal distribution for the fitness of the isolates, in agreement with the hypothesis of a polygenic quantitative trait.

**Fig. 4:**
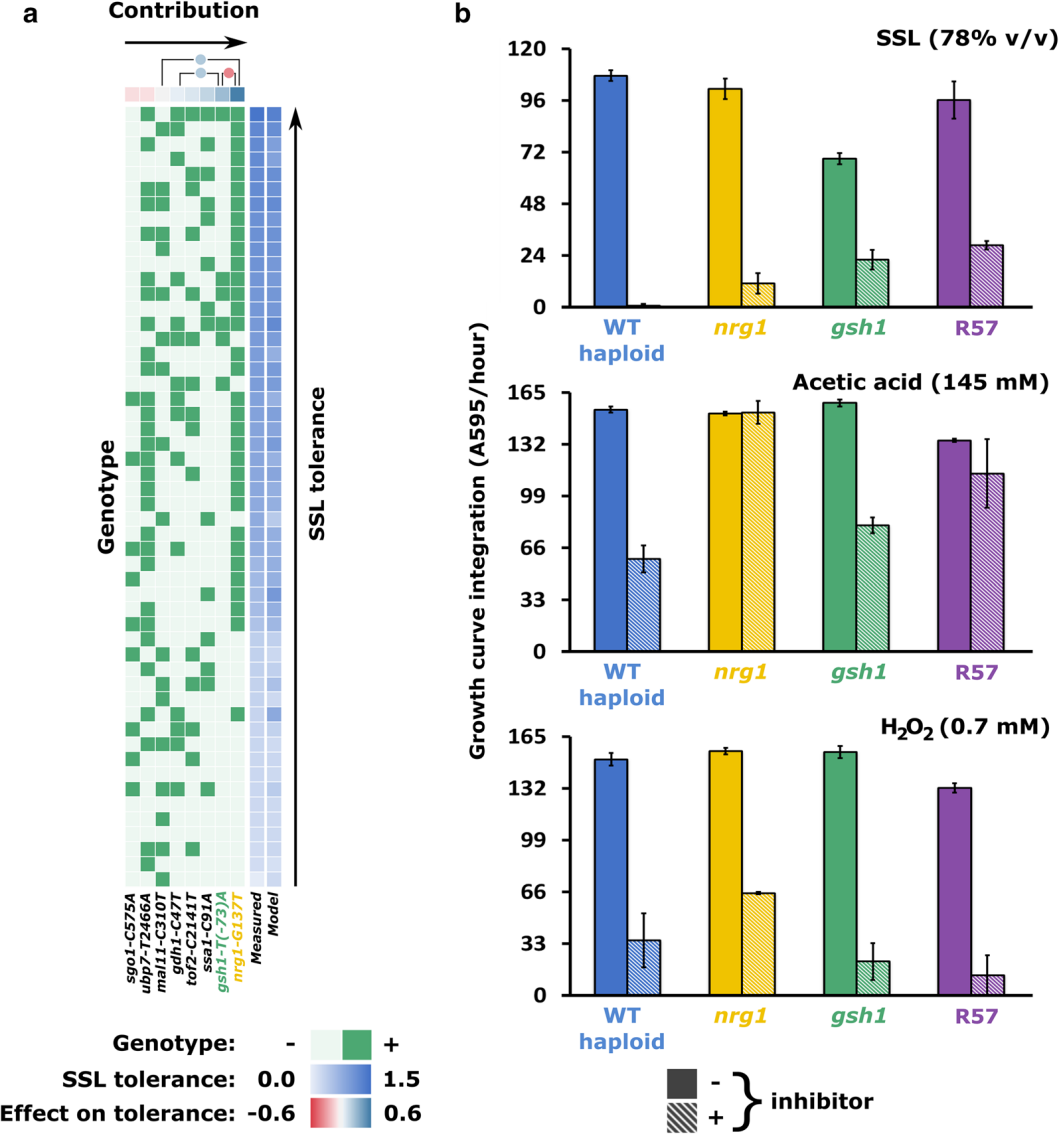
Mutations in *NRG1* and *GSH1* are the main determinants of hydrolysate tolerance. Multiple linear regression (**a**) on the genotypes of segregants of an SSL-tolerant mutant identifies mutations *nrg1*-*G137T* and *gsh1*-*T(*-*73)A* as the strongest predictors of high tolerance. This prediction is confirmed in point mutants (**b**), with both mutations conferring increased tolerance to SSL. The *nrg1*-*G137T* mutation also increases tolerance to acetic acid and hydrogen peroxide in haploids. Bars in the graphs report area under the growth curve for the indicated mutants recorded in the presence (solid bars) and absence (striped bars) of inhibitors. Error bars represent ± 1 standard deviation

We used amplicon sequencing to genotype the 86 segregants. For the vast majority of strains and loci, we achieved depth of coverage well above 30 with an average of 794, enabling confident genotyping (Additional file 1: Table S2). Multiple linear regression analysis was applied separately on the haploid and diploid data sets to predict the effect of each SNP on the phenotype (summarized in Fig. 4a, Additional file 7: Figure S4). Fewer variables and more data points mean that we have higher confidence in the haploid model than in the diploid model (see Additional file 5). Accordingly, there is better agreement between predictions of the haploid model and measured phenotypes. In both haploids and diploids, the strongest predictor for enhanced tolerance is the *nrg1*-*G137T* mutation. Examination of the genotype heatmap in Fig. 4a shows a clear clustering of *nrg1* isolates at higher inhibitor tolerance levels. In haploids, the *gsh1*-*T(*-*73)A* mutation is also strongly associated with a high tolerance phenotype, although the model proposes negative epistasis between the *nrg1* and *gsh1* alleles. Mutations *ssa1*-*C91A*, *tof2*-*C2141T* and *gdh1*-*C47T* are also associated with modest increases in haploid tolerance, albeit the effect of the latter is proposed to be enhanced by interaction with *gsh1*-*T(*-*73)A*. Deleterious for haploids are mutations *mal11*-*C310T*, *ubp7*-*T2466A* and especially *sgo1*-*C575A*. Mutation *mal11*-*C310T* has virtually no effect (*p* value = 0.254 that linear coefficient is equal to 0), but is proposed to interact with *nrg1*-*G137T* to further increase tolerance.

### Reconstruction and reversion of SNPs

To confirm the contribution of individual mutations to the tolerance phenotype, a subgroup of 27 was reconstructed into the *MATα* parental strain. In agreement with our backcross experiment, *gsh1*-*T(*-*73)A* and *nrg1*-*G137T* both increased fitness of wild-type haploids (Fig. 4b). In contrast, the same SNPs in hetero- and homozygous wild-type diploids did not increase fitness (Additional file 8: Figure S5). Fitness of haploid and diploid *nrg1 gsh1* double mutants is not higher than that of the fittest mutant, suggesting that the effect of these SNPs is non-cumulative. Further supporting a contribution of these SNPs to the phenotype, reversion to wild-type abolished the enhanced phenotype of haploid single mutants (Additional file 9: Figure S6).

To narrow the physicochemical stresses to which *nrg1* and *gsh1* mutations conferred resistance, we compared the fitness of haploid single mutants with wild type and R57 cells exposed to acetic acid or hydrogen peroxide (Fig. 4b). The *nrg1*-*G137T* mutation bestowed increased fitness in the presence of both compounds, enabling faster growth than wild type and R57. The *gsh1*-*T(*-*73)A* mutation does not seem to confer the same advantage. Among mutations identified by population sequencing, a subset of the most positively selected was also introduced in the *MATα* haploid, but an increase in tolerance was not observed for those strains.

Each SNP was also reverted to wild type in strain R57 (Additional file 10: Figure S7A). At most loci, reversion did not cause a detectable loss of fitness. The only exception was reversion of the *gdh1*-*G47A* mutation, which led to a pronounced decrease in fitness in the R57, R57 *GDH1*-*68* and R57 *SGO1* backgrounds. The loss of fitness associated with reversion of *gdh1* alleles in the R57 background contrasts with the deleterious effect of these mutations in wild-type backgrounds (Fig. 3) and indicates reciprocal sign epistasis. We hypothesized that *gdh1* alleles complement secondary deleterious mutations found in R57. We further reverted single mutations to wild type in the *GDH1* or *SGO1 gdh1*-*T68G* backgrounds to identify those that would rescue the growth defects. The R57 *GDH1* growth defect could not be rescued by reversion of single secondary SNPs. While we could not fully reproduce the R57 *SGO1 gdh1*-*T68G* growth defect, possibly because of batch-to-batch variations in hydrolysate composition, we identified two double revertants with reduced tolerance (Additional file 10: Figure S7B). Removal of mutation *ynl058c*-*A7G* in this background confers wild-type tolerance to SSL. Consistent with other observations, reversion of *nrg1*-*G137T* also leads to a loss of tolerance in the R57 *SGO1 gdh1*-*T68G* background. This effect is not observed in the R57 *GDH1* background because loss of both *gdh1* alleles leads to a loss of fitness that is too important to observe the effect of reverting to *NRG1* or *YNL058C*. The partial loss of fitness in the homozygous *gdh1*-*A68G* derivative of R57 leaves room to observe the additional effect of reverting other mutations. Together, these results show that *gdh1* alleles exert their effect via an epistatic mechanism. The precise mechanism is still elusive, but from the growth defect of R57 *GDH1* cells in the presence and absence of inhibitors, we speculate that they act by complementation in a complex network of genetic interactions.

## Discussion

The results of this study identified key molecular determinants of hydrolysate tolerance. Our evidence identifies genes *NRG1*, *GSH1* and *GDH1* as having the strongest impact on the selected phenotype. Below, we discuss their significance along with hypotheses on the contribution of other mutant alleles identified in this study. Knowledge on the genetic architecture of inhibitor-tolerant mutants also informs our understanding of the evolutionary determinants that dictated the outcomes of our genome shuffling experiment. Further below, we discuss these evolutionary determinants and how they may impact the outcomes of genome shuffling experiments.

### Molecular determinants of fitness in lignocellulosic hydrolysates

Our evidence indicates that the low frequency but strongly selected mutation *nrg1*-*G137T* confers the strongest direct gains in hydrolysate inhibitor tolerance among all SNPs considered in this study (Fig. 4, Additional file 4: Figure S2, Additional file 7: Figure S4, Additional file 9: Figure S6 and Additional file 10: Figure S7). These results are consistent with previous RNAseq results in R57 that showed considerable upregulation of five targets of transcriptional regulator Nrg1p, including *NRG1* itself [6].

*NRG1* and its close paralog *NRG2* encode DNA-binding proteins first identified as mediators of glucose repression [26, 27]. The Nrg1/2p repressors have been implicated in the response to various stresses, including glucose [28] and zinc limitation [29], alkaline pH [30], salt tolerance [31, 32] and organic acid challenge [33]. Identification of transcripts with altered expression in null mutants of *NRG1* and *NRG2* established their role in the regulation of the general stress response [34]. Deletion of *NRG1* or *NRG2* changes the transcription of 150 genes, many of which display stress response elements (STREs) or related sequences in their promoter regions. A significant overlap between the Nrg1/2p and Msn2/4p regulons further supports a role for Nrg proteins in the regulation of the general stress response [26, 35]. We, therefore, propose that *nrg1*-*C137A* is a loss of function mutation that leads to the upregulation of general stress response genes.

Our models predict that mutation *gsh1*-*T(*-*73)A* makes the second largest contribution to inhibitor tolerance in haploids (Fig. 4a). Accordingly, haploids carrying this single mutation display enhanced growth in the presence of high concentrations of hydrolysate **(**Fig. 4b). The near sweep of the *MATα* mutant pool by the *gsh1*-*T(*-*73)A* allele also suggests a significant selective advantage in the presence of high concentrations of hydrolysate. We have previously shown that haploids carrying the mutant *gsh1*-*T(*-*73)A* allele accumulate lower levels of reactive oxygen species (ROS) than their wild-type parents when exposed to high concentrations of hydrolysate [36]. This is consistent with the role of Gsh1p in the synthesis of the antioxidant glutathione [37]. The mutation identified in our mutants is located 73 bp upstream of the start codon, outside Yap1p and other hydrogen peroxide responsive sequences [38–40]. The position of this SNP in the region proximal to the start codon identifies alteration of the basal promoter as the most likely mechanism. We propose that upregulation of *GSH1* by this modified promoter increases glutathione synthesis and reduces accumulation of ROS in *gsh1*-*T(*-*73)A* mutants.

Several lines of evidence point to *GDH1* as a key determinant of selection in our genome shuffling experiment. Notably, this gene is the most populated mutation hotspot (Fig. 3a) and *gdh1* alleles appear required for hydrolysate tolerance in mutant R57 (Fig. 3d). However, their effect appears epistatic, since their introduction into wild-type backgrounds is associated with growth defects, perhaps suggesting a compensatory role in hydrolysate tolerant strain R57 (Fig. 3d).Glutamate dehydrogenase Gdh1p, along with close homolog Gdh3p, catalyzes amination of α-ketoglutarate, yielding glutamate [41]. Under fermentative conditions, Gdh1p is the dominant glutamate dehydrogenase, while carbon limitation, non-fermentable carbon sources and entry into the stationary phase induce the expression of Gdh3p [42, 43]. Transcription of *GDH1* is sustained at all phases of growth, but entry into the stationary phase triggers ubiquitin-mediated degradation of Gdh1p. Gdh3p is specifically expressed during the stationary phase. Gdh1p is a faster enzyme, suited for growth-sustaining glutamate synthesis. Gdh3p is slower, better suited to sustain glutathione synthesis during the stationary phase and under stressful conditions [42]. Accordingly, Gdh3p has been implicated in stress tolerance in yeast, while transient loss of tolerance to hydrogen peroxide is observed during early phases of growth in *gdh1Δ* mutants [43]. From the mapping of the seven amino acid substitutions on the Gdh1 protein, we suggest two potential mechanisms for their action. One possibility is that they affect inter-domain flexibility and, thus, catalytic activity, as suggested by structural studies of bacterial homologs [44]. The substitutions are located near known ubiquitination sites [43, 45]. A second hypothesis is thus that they impair degradation of Gdh1p in conditions of stress or during the stationary phase. Regardless of its underlying mechanism, we propose that the *gdh1* hotspot was selected to compensate the pull on glutamate exerted by upregulation of glutathione biosynthesis in *gsh1*-*T(*-*73)A* mutants, especially under hydrolysate inhibitor stress. This hypothesis would explain the strong growth defect incurred by R57 upon reversion of *gdh1* mutations and, therefore, the reciprocal sign epistasis observed with these mutations.

The role of *MAL11* in the transport of the stress-protectant molecule trehalose suggests involvement of mutations of this hotspot in hydrolysate tolerance [46]. Our model of hydrolysate tolerance in haploids suggests interactions between the *nrg1*-*C137A* and *mal11*-*C310T* mutations. *MAL11* is repressed by glucose, notably via the action of Mig1p [47–49]. Overlap between the Mig1p and Nrg1p regulons suggests a potential mechanism for this regulation [26].

Reversion of the *ynl058c*-*A7G* mutation in R57 does not cause a detectable loss of tolerance to the hydrolysate inhibitors. However, in a derivative of R57 wild type at the *SGO1* locus and homozygous for the *gdh1*-*A68G* allele, reversion to wild type at the *YNL058C* locus leads to a loss of SSL tolerance. This suggests a role for this gene in inhibitor tolerance. The function of *YNL058C* is essentially unknown, but the protein it encodes appears to localize to the vacuole [50]. Both *YNL058C* and its paralog *PRM5* are induced via the cell wall integrity pathway, indicating a role in the response to cell wall damage [51, 52]. Downregulation of *YNL058C* was also observed upon DNA damage [53]. Together, these reports indicate a role for *YNL058C* in the response to stress and cell damage, in agreement with an involvement in hydrolysate tolerance.

Mutation *ste5*-*C512T* displays the highest apparent selection and average frequency in cohort **a6**. The model of R57 backcrosses indicates that it makes the second highest contribution to inhibitor tolerance in diploids (Additional file 7: Figure S4). *STE5* encodes a scaffold protein involved in facilitation and integration of pheromone-induced MAPK signaling [54]. Components of the pheromone-induced MAPK pathway were also implicated in stress signaling [55–59]. Because Ste5p binds the Kss1p MAPK, it mediates signals involved in stress. It is thus likely that the *ste5*-*C512T* mutation modulates the Ste20p-Ste11p-Ste7p-Kss1p pathway to stimulate the execution of a stress response program.

**Fig. 5:**
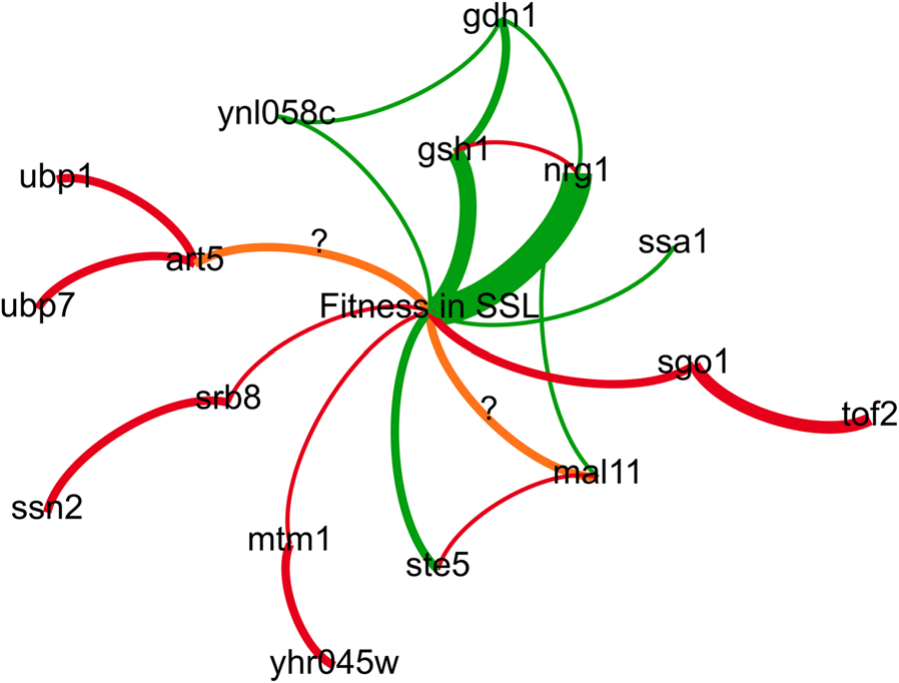
Network map model of SSL tolerance in genome shuffled mutants. Nodes represent mutations or phenotypes and edges represent effects or interactions. Curvature of the edges is clockwise with respect to effector nodes and counter-clockwise with respect to target nodes. Green edges indicate a stimulating or enhancing effect, while red lines indicate an inhibition of the target. Orange lines indicate persisting doubts on the relationship between the nodes that they connect. Thickness of the edges is related to the amount of evidence available for the indicated interactions

Our understanding of the hydrolysate inhibitor tolerance phenotype selected in our genome shuffling mutants, as described above, is summarized in Fig. 5. In brief, our results indicate that mutant alleles *nrg1*-*G137T* and *gsh1*-*T(*-*73)A* are the main determinants of hydrolysate inhibitor tolerance in our evolved mutants. Our evidence also ascribes a critical role for *gdh1* alleles. The phenotype associated with these alleles in parental and mutant strains demonstrates reciprocal sign epistasis and is coherent with a compensatory mechanism. We propose putative mechanisms for other potential contributing alleles. These alleles may help in the rational engineering of hydrolysate inhibitor tolerance strains. However, a recent study showed that the genetic determinants of hydrolysate inhibitor tolerance in yeast can prove highly strain-dependent [60]. Therefore, it would be interesting to test the impact of mutations identified herein in various industrial strain backgrounds.

### Evolutionary determinants of selection by genome shuffling

Our experiment is characterized by a set of early genetic features that had a determining effect on its overall outcomes. These features are the presence of mutations in the parental strains, prevalent genetic hitchhiking, and a selective sweep that severely reduced diversity at early selection steps. We argue that these features favored specific evolutionary solutions and elicited a strong selective pressure in favor of compensatory mechanisms.

The *MATα* parental strain contained mutations *mtm1*-*A943T*, *avl9*-*C1806G* and *sro77*-*G(*-*160)T*, while the *MATa* parent carried SNPs *srb8*-*C3787G* and *art5*-*G454T*. We draw parallels between these founding mutations and mutational hotspots identified by population genome sequencing. Both *MTM1* and *YHR045W* have been implicated in iron metabolism [61–63]. Similarly, the *ssn2* hotspot can be linked to the *srb8*-*C3787G* substitution. Both genes encode subunits of the RNA polymerase II mediator complex [64–68]. The *ubp1* hotspot is notable, owing to the high frequency of the *ubp7*-*T2466A* mutation in our pool. Both genes encode ubiquitin-specific proteases [69, 70]. The prevalence of the founding *art5*-*G454T* mutation, mapping to a gene involved in the regulation of membrane protein homeostasis [71], suggests that complementing mutations involved in this cell process were selected during our experiment. The *yhr045w*, *ssn2* and *ubp1* mutations all remain at low frequency, with either weakly positive or negative apparent selection. This would suggest that complementation of founder mutations confers marginal competitive advantages. However, the prevalence and persistence of mutation *ubp7*-*T2466A* are hypothesized to result from the same epistatic dynamics. The coincidental presence of this mutation in the same mutant as the **α3** driver *gsh1*-*T(*-*73)A* likely caused the selection of *ubp7*-*T2466A* by hitchhiking, while its arguably minor role in complementing *art5*-*G454T* was seemingly sufficient to ensure its persistence, in contrast with other hitchhikers of the same cohort.

Clustering of mutations in cohorts with correlated evolutionary trajectories is commonly observed in experimental evolution experiments as a signature of genetic hitchhiking [14]. In the context of our genome shuffling experiment, this observation suggests that a large proportion of mutant alleles arose together in a few founding individuals. Prominent examples are cohorts **a5**, **a6** and **α3**, putatively driven by *ste5*-*C512T* or *gdh1*-*G299A*, and *gsh1*-*T(*-*73)A*, respectively (Fig. 2). We expect that most examples of genetic hitchhiking negatively affected the fitness of mutants, because the majority of mutations tend to be neutral or deleterious [72]. Previous studies in yeast on the evolutionary effects of sexual recombination suggest that it favors purifying selection of hitchhikers [16]. Accordingly, the majority of putative hitchhikers in our experiment either display consistently low frequency or negative apparent selection (Fig. 2). Despite evidence of purifying selection, putative hitchhikers of cohort **α3** persist at high frequency until the end of the GS experiment. This could in part be due to vegetative cells escaping the sporulation and mating process. Yet, sporulation efficiency was high (40% on average) and both digestion and sonication of vegetative cells after sporulation should have minimized the impact of non-shuffled mutants on the evolutionary dynamics of the experiment. Further, cohort **α3** nearly swept the initial pool of *MATα* mutants likely due to an aggressive early selection. This first selection was performed before the onset of sexual recombination cycles and illustrates the well-documented effect of clonal interference on the evolution of asexually reproducing populations [14, 16].

We propose that the founding features discussed above caused major evolutionary responses, and that those are critical to understanding the dynamics of the experiment. For example, the presence of mutational hotspots identifies loci under strong selective pressure and we have shown that many appear related to founding mutations. Similarly, the mechanism proposed above for epistasis between *gdh1* and *gsh1* mutant alleles would explain the evolutionary signal detected at the *GDH1* hotspot. The convergence of the *gsh1*-driven selective sweep with founding mutation *art5*-*G454T* also appears to have driven the persistence of the *ubp7*-*T2466A* hitchhiker allele. Finally, the minor evolutionary role played by the tolerance-conferring *nrg1*-*G137T* mutation may be caused at least in part by the founder effect favoring *gsh1*-*T(*-*73)A* coupled to negative epistasis between the two alleles, as suggested by our linear model of hydrolysate tolerance (Fig. 4a). This observation resonates with experimental evolution studies of sexually reproducing yeast that showed a major role for standing diversity and ascribed a minor evolutionary role to rare or de novo mutations [23]. It also illustrates the dominant role played by compensatory mechanisms over direct enhancement of hydrolysate tolerance in our experiment.

In contrast to previous experimental studies of compensatory evolution, the examples proposed in this study rely on convergent signals at specific loci. Convergent compensatory evolution at the functional rather molecular level has previously been reported [73]. Because the path of compensatory evolution was shown to be constrained both by environmental factors and the genetic background, the level of molecular convergence that we observe would indicate a targeted response to highly specific conditions [74].

### Descriptive model of evolutionary dynamics in the genome shuffling experiment

Based on the proposed model of SSL tolerance and on the allele frequency time series obtained from population sequencing (Fig. 2), we propose a model to describe the evolutionary dynamics of our genome shuffling experiment. Mutagenesis generated equally diverse pools of mutants from the *MATα* and *MATa* parental strains. An aggressive selection regimen restricted genetic diversity in the *MATα* pool, leading to a near sweep by mutants carrying the *gsh1*-*T(*-*73)A* allele and **α3** hitchhikers. Mutations *mal11*-*A482T* and *gdh1*-*A68G* were found at a low frequency in this pool. A more relaxed selection generated a more diverse pool of *MATa* mutants, among which were tolerance enhancing *nrg1*-*C137A* and *ste5*-*C512T* mutations. Cohorts **a6** and perhaps **a5** hitchhiked on this latter mutation. The *gdh1* mutations (with the exception of A68G) and *mal11*-*G310A* were also selected into this initial pool. Initial recombination created the first epistatic pairs between tolerance-conferring and compensatory mutations. Combinations of founders with their complementing mutations also occurred on a large scale at this stage. Selection on these shuffled mutants brought a large increase in the frequency of complementing mutations; thanks to the competitive advantage they imparted onto SSL-tolerant but metabolically imbalanced mutants. Further shuffling and selection brought several of these epistatic relationships into single cells, increasing their fitness in the presence of SSL. The strong selective advantage of *nrg1*-*C137A* and perhaps its separation from deleterious alleles to which it was initially linked led to its steady increase in frequency from a low initial frequency. Additional rounds of shuffling could have witnessed the rise to prominence of this *nrg1* allele.

## Conclusions

In this study, we identified some of the key determinants of selection in a genome shuffling experiment. We ascribed critical roles to loci *NRG1*, *GSH1* and *GDH1* to the hydrolysate inhibitor tolerance phenotype. With regard to recent results [60], the applicability of these evolutionary solutions to different strain backgrounds remains to be established.

More fundamentally, we have illustrated the impact that a few, early features can have on the course of evolutionary engineering by genome shuffling. It also suggests that this regimen of recursive recombination leads to the widespread selection of compensatory mechanisms, illustrating the construction of strains in which delicate complementation networks operate to offset the fitness cost incurred by founding mutations, hitchhikers and the pleiotropic effects of core beneficial alleles. To expand on the specific outcomes of our experiment, we propose that purposefully designed genome shuffling experiments performed on diverse genetic backgrounds with precise variations in initial conditions could aid the design of future strain development endeavors, and contribute to our understanding of evolution in sexually reproducing populations, especially as it pertains to compensatory evolution.

## Methods

### Evolution by genome shuffling

All strains of *S. cerevisiae* used in this study are derived from prototrophic strains CEN.PK113-1A (wild-type *MATα*) and CEN.PK113-7D (wild-type *MATa*). Mating of the haploids was used to generate the wild-type diploid. The genome shuffling experiment is described in detail in a previous publication [24] and is summarized in Fig. 1. Briefly, to generate genetically diverse starting populations, *MATα* and *MATa* haploid mutant pools were generated by UV irradiation. These initial pools were spread onto gradient plates for selection of cells with inhibitor tolerance above the wild type. Irradiated cells growing above the wild type were scraped off the plate and aliquots from both populations were preserved as glycerol stocks at − 80 °C for later sequencing. The two populations of selected haploid were mixed 1:1 for mating and diploids were selected on gradient plates. This first population of selected diploids was sporulated, digesting vegetative cells with Zymolyase (MP Biomedicals) and sonicating them before mating, generating a first genome shuffled population R1. Four additional recursive cycles of selection, sporulation and mating were performed resulting in populations R2–R5, which showed increasing tolerance to the inhibitory substrate at each mating cycle. Genome shuffled populations selected on gradient plates were propagated overnight in YPD at 30 °C and aliquots were preserved as glycerol stocks at − 80 °C for later sequencing.

### Pool-seq of evolved populations

Prior to genomic DNA extractions, cells from glycerol stocks of the two UV irradiated (a-UV and α-UV) and five genome shuffled populations (R1–R5) were thawed and incubated in YPD for 1 h. They were then suspended in 50 mM Tris–HCl pH 8.0,10 mM EDTA, 5% 2-mercaptoethanol (v/v), 200 U/ml yeast lytic enzyme (MP Biomedicals) for 1 h at 37 °C. Genomic DNA was extracted using the DNeasy Blood and Tissue Kit (Qiagen) according to the manufacturer’s instructions and quantified using the QuantiFluor dsDNA System (Promega). Genomic DNA was sequenced at the McGill University and Genome Quebec Innovation Centre using the TrueSeq library preparation reagents and an Illumina HiSeq 2500 sequencer (100 bp paired-end reads). Each of the 7 populations was sequenced on a separate lane of a HiSeq chip to maximize depth of coverage.

Quality control of raw sequencing data was performed using FastQC [75] and overlapping read pairs were merged with PEAR [76]. Alignment to the CEN.PK113-7D reference genome [25] was done using *bwa mem* [77] and performed separately for overlapping and non-overlapping reads. Output SAM files for overlapping and non-overlapping reads were merged with the MergeSamFiles utility in Picard Tools [78]. Picard was next used to add read groups, sort reads, then mark and remove duplicates prior to indel realignment with the Genome Analysis Toolkit [79–81]. Alignment metrics were extracted using Picard Tools.

SNPs were called and filtered using a base error model inspired from Barrick and Lenski [18] as detailed in Additional file 5.

### Mutant allele frequency, strength of selection and hierarchical clustering of evolutionary trajectories

The proportion of mismatch reads at given genomic coordinates was considered to reflect the frequency (*p*) of the associated mutant allele within the sequenced populations. The frequency of all mutations was extracted for the seven sequenced populations. The frequency of each mutation was necessarily zero in one of the haploid populations, allowing the identification of its origin (*MATα* or *MATa*). Furthermore, because the two haploid populations represented a single evolutionary time point, their frequency was averaged to obtain the pre-shuffling allele frequencies (i.e., *p*_UV_ = (*p*_α_ + *p*_a_)/2). Allele frequencies of the R1–R5 populations each represented time points of their own.

Strength of the positive or negative selection was estimated from allele frequency change across the time points (Δ*p*_t1−t2_ = *p*_t2_/*p*_t1_). The geometric mean frequency change (noted M) was used as a synthetic measure of selection to smooth the effect of proportionally large frequency changes often observed between time points UV and R1. Hierarchical clustering of evolutionary trajectories was performed by running the clustermap routine of the Seaborn Python library [82].

### Backcrossing experiment

To assess the contribution of each SNP to the inhibitor tolerance phenotype, strain R57 was backcrossed with parental wild-type *MATα*. Cells from the resulting F_2_ population were scored for tolerance to inhibitors (Additional file 6: Figure S3). Haploids of R57 were generated on sporulation medium (1% potassium acetate, 0.1% yeast extract, 0.05% dextrose, 2% agar) incubated at room temperature for 6 days, resulting in > 50% sporulation efficiency. Cells were scraped from the plate and digested overnight at 30 °C with 100 U of yeast lytic enzyme (MP Biomedicals) in 5 ml of H_2_O containing 10 μl of 2-mercaptoethanol. Five microlitre of 1.5% IGEPAL was added to the digest, incubated on ice for 15 min and sonicated 3× for 30 s. Spores were harvested by centrifugation at 12,000×*g* for 10 min and suspended in 5 ml 1.5% IGEPAL. The sonication procedure was repeated once more and the spores were suspended in 250 μl of YPD broth. For mating, spores were mixed with an approximately equal amount of *MATα* cells (CENPK113-1A), spotted on YPD agar and incubated overnight at 30 °C. Resulting R57 × 1A spores were spotted on YPD agar and then allowed to germinate and mate for 48 h. The resulting diploids were submitted to a second round of sporulation and mating to further shuffle mutations. After mating, cells were streaked for single colonies on YPD agar and 86 isolated colonies were picked for genotyping and fitness assays.

### Preparation of yeast genomic DNA template for PCR

Cells from 1.5 ml of culture were harvested in a tabletop microcentrifuge and the resulting pellet was suspended in 250 μl of 50 mM Tris–Cl pH 8.0 supplemented by 20 U of yeast lytic enzyme. This digestion solution was incubated at 37 °C for 1 h. Cell lysis was induced by adding 250 μl of 200 mM NaOH, 1% SDS, vortexing, and incubating for 5 min at room temperature. The lysates were neutralized with the addition of 350 μl of 3 M potassium acetate pH 5.5 and clarified by centrifugation at 13,300×*g* for 10 min. DNA from the resulting supernatants was precipitated by adding 600 μl of 2-propanol and centrifuged at 13,300×*g* for 10 min. The resulting DNA pellet was air dried for 15 min, suspended in 100 μl of H_2_O, and vortexed before incubation for 15 min in a water bath at 55 °C.

### Genotyping of backcrossed isolates

We genotyped 86 backcrossed isolates at 18 of the mutant loci identified in R57 by PCR amplicon sequencing. Both forward and reverse primers consisted of a common 5′ heel sequence (forward: 5′-CGTTCAACCTTGTCCAACAGTG-3′ and reverse: 5′-GAAGCGATGACTCGAGCGTATT-3′) and a 24–28 nucleotide gene-specific sequence at the 3′ end. PCRs contained genomic DNA template, 0.5 μM primers, 200 μM dNTPs, 1× high fidelity Phusion buffer (Thermo Fisher), 1.5% DMSO and 1 U of Phusion High Fidelity DNA polymerase (Thermo Fisher) in 50 μl. Cycling conditions were as follows: 98 °C for 30 s, then 35 cycles of 98 °C for 10 s, 55 °C for 20 s, 72 °C for 4 s, followed by a 5 min final extension at 72 °C. Ion torrent sequencing adapters and barcodes were added in a second PCR. This second reaction was performed using a common reverse primer consisting of the ion torrent P1 adapter (5′-CCTCTCTATGGGCAGTCGGTGAT-3′) and the reverse heel sequence. The 96 forward primers consisted of the ion torrent A adapter (5′-CCATCTCATCCCTGCGTGTCTCCGACTCAG-3′), a unique 8 nucleotide barcode and the forward heel sequence. Composition of this second PCR was the same as for locus-specific amplification, using 1 μl of a 1:10 dilution of the first reaction as template. Cycling conditions were: 98 °C for 30 s, then 35 cycles of 98 °C for 10 s, 70 °C for 20 s, 72 °C for 6 s, followed by a 5 min final extension at 72 °C. All primers used for genotyping are listed in Additional file 1: Table S3. PCR products carrying the same barcode were pooled and DNA migrating between 100 and 400 bp was purified on a 2% agarose gel using the GeneJet gel extraction kit (Thermo Fisher). Equal amounts of all pools were mixed to make the sequencing library. The DNA concentration of this pool was measured on a Qubit 2.0 fluorometer (Life Technologies). Ion torrent sequencing template was prepared from our pooled library with an Ion PGM Template OT2 400 Kit according to the manufacturer’s instructions. Sequencing was performed with an Ion PGM sequencer using the Ion PGM Hi-Q sequencing kit and an Ion 316 Chip v2 BC, all following manufacturer’s protocols and instructions.

Reads from the Ion PGM were sorted and trimmed using a custom python script, and then aligned to the CEN.PK113-7D reference genome [25] using bwa mem [77]. Read counts for each of the SNP positions were extracted with bam-readcount [83] setting both minimum mapping quality and minimum base quality at 30. Genotypes were called from read counts as follows: heterozygotes were distinguished from homozygotes by assuming that in homozygotes, the most frequent base call would have a frequency of 0.997 and all other base calls 0.001 each. A *G* test was performed to test whether the base count distribution differed significantly from this assumption. If it did, a heterozygous genotype was called. Otherwise, the identity of the most frequent base call was checked. If the most frequent base call was the reference base, a wild-type genotype was called, otherwise a homozygous mutant genotype was called. In strains previously identified as haploid, heterozygosity calls were corrected and genotype was called based on the identity of the most frequent base call. All genotype calls were reviewed visually.

### Reconstruction and reversion of SNPs using CRISPR-Cas9

To probe their contribution on the observed phenotype, point mutations were reintroduced in wild-type backgrounds (CEN.PK113-1A, *MATα* or CEN.PK113-7D, *MATa*) or reverted to wild type in strain R57 using CRISPR-Cas9 as described previously [36]. Briefly, gRNAs were designed to introduce double-stranded breaks (DSBs) in the vicinity of the mutation. DSBs were repaired by homologous recombination with donor DNAs replacing the 20-nt protospacer sequence by a heterologous stuffer sequence. This stuffer was targeted by a second gRNA and the resulting DSBs were repaired by a stuffer-free donor DNA carrying the point mutations. The result was the seamless introduction of single nucleotide changes.

### Fitness assays

Cell growth when exposed to varying concentrations of the inhibitory substrate was used to measure the fitness of the strains. Prior to the assays, cells we pre-adapted with overnight incubations in undiluted inhibitory substrate at 30 °C. For fitness assays, adapted cells were washed 3× in 10 mM sodium citrate pH 5.5 and inoculated to a final concentration of ~ 2 × 10^5^ into YNB 1% glucose supplemented with varying concentrations (0–85%) of the inhibitory substrate in 96-well plates (Costar 3595, Corning). Plates were incubated at 30 °C with shaking in a Tecan Sunrise absorbance reader, measuring absorbance at 595 nm every 20 min. Because inhibitors may affect growth rate, lag time, maximum cell density or any combination thereof, we used the area under the growth curves as a measure of fitness. The lignocellulosic inhibitory substrate was kindly supplied by Tembec (Témiscaming, Québec) and AV Cell (Atholville, New Brunswick). The pH of the substrate was adjusted to 5.5 with 10 M NaOH prior to use in culture medium.

### Multiple linear regression model

Detailed description of multiple linear regression methodology is provided in Additional file 5. Briefly, contribution of individual SNPs to the phenotype was predicted from linear models. These models were built by multiple linear regression, using genotype at each of the mutant loci as explanatory variables and area under growth curves in 85% inhibitor substrate as the response variable. Distinct models were built for haploid and diploid mutants. A single binary variable was used for each locus in haploids, with wild type = 0 and mutant = 1. In diploids, each locus was represented by three binary variables representing the wild type, heterozygous mutant and homozygous mutant genotypes, respectively. For the haploid model, fitting using all possible combinations of variables was performed and the model that minimized Mallows Cp and variance of residuals was chosen. The large number of variables involved with diploids made this approach impractical and so it was performed in a stepwise manner. Interaction between loci was modeled in an ad hoc manner, using a stepwise methodology similar to what was used for diploids.

## Additional files

**Additional file 1: Table S1.** Population sequencing and alignment metrics. **Table S2.** Amplicon sequencing and alignment metrics for genotyping of R57 backcrossing isolates. **Table S3.** List of primers used for production of Ion Torrent sequencing libraries.

**Additional file 2.** List of single nucleotide polymorphisms detected by whole population sequencing.

**Additional file 3: Figure S1.** Evolutionary trajectories for all non-silent mutations identified by population genome sequencing at 6 time points. Mutations arose either in the *MATα* (left) or *MATa* (right) haploid populations. On the vertical axis are the names of the mutations, giving the closest gene, coordinates with respect to that gene and the nature of the nucleotide substitution. On the horizontal axis are each of the six evolutionary time points (UV, R1, R2, R3, R4, R5) and the mean allele frequency change (M). Frequencies of the mutant alleles are represented by shades of green. Mean allele frequency changes are represented in shades of red (M < 1, declining frequency) or blue (M > 1, increasing frequency). Hierarchical clustering of individual evolutionary trajectories is represented by dendrograms on the left. Mutations were assigned to groups of mutations (a1-5, α1-4) on the basis of this clustering. Mutations present in highly tolerant mutant R57 are highlighted in bold.

**Additional file 4: Figure S2.** Evolutionary trajectories and apparent selection of all mutation hotspots identified by population sequencing. Mutations arose either in the *MATα* (left) or *MATa* (right) as indicated immediately to the left of each mutation. On the vertical axis are the names of the mutations, giving the closest gene, coordinates with respect to that gene and the nature of the nucleotide substitution. On the horizontal axis are each of the six evolutionary time points (UV, R1, R2, R3, R4, R5) and the mean allele frequency change (M). Frequencies of the mutant alleles are represented by shades of green. Mean allele frequency changes are represented in shades of red (M < 1, declining frequency) or blue (M > 1, increasing frequency). Mutations linked by connectors and marked with an asterisk indicate pairs with significantly similar initial frequency (binomial test, p > 0.05).

**Additional file 5:** Supporting methods. Detailed methods are provided for our SNP calling methodology, structural study of Gdh1p, determination of mating type and ploidy by PCR, and modeling of SSL tolerance by multiple linear regression.

**Additional file 6: Figure S3.** Backcrossing of R57 with wild type cells generates strains presenting a wide spectrum of fitness in SSL. Growth in the presence and absence of SSL is reported for R57, various wild type cell types and 86 F2 isolates from backcrossing of R57 and CEN.PK113-1A. Error bars represent plus or minus one standard deviation. The dashed line is a visual reference for the level achieved by the wildtype.

**Additional file 7: Figure S4.** Genotyping of second generation segregants from backcrossing of R57 and wild type yeast suggest a model of SNP contributions to the SSL tolerance phenotype. Haploid (A) and diploid (B) isolates are scored in green for the genotypes indicated at the bottom. Growth in 85% SSL is scored in shades of blue on the right. Each row represents a single strain. Contribution to the phenotype of the indicated genotypes was inferred by multiple linear regression, yielding coefficients represented at the top in shades of red (diminishes fitness) to blue (increases fitness). Modeling of genetic interactions was attempted and the resulting coefficients are represented as circles at the top of the heatmaps. Growth in SSL predicted by the linear model is reported in shades of blue in the rightmost column, showing the level of agreement between the model and the data.

**Additional file 8: Figure S5.** Single mutations are not sufficient to detect an increase in SSL tolerance in diploid cells. Area under the growth curve in the presence and absence of SSL for heterozygous (A) and homozygous (B) single diploid mutants is reported. Error bars represent plus or minus one standard deviation. The dashed line is a visual reference for the level achieved by the wildtype.

**Additional file 9: Figure S6.** Reversion of *nrg1* and *gsh1* mutations leads to loss of the SSL tolerance phenotype in haploid single mutants. Area under the growth curve for *nrg1* and *gsh1* double mutants, haploid (*nrg1 gsh1*) and diploid (*nrg1*/*nrg1 gsh1*/*GSH1*) is also reported. Error bars represent plus or minus one standard deviation. The dashed line is a visual reference for the level achieved by the wildtype.

**Additional file 10: Figure S7.** Growth in the presence and absence of SSL of single and double revertants identifies mutations contributing to the SSL tolerance phenotype. Area under the growth curve in the presence and absence of SSL is reported for (A) single haploid mutants, (B) single revertant derivatives of R57 and (C) revertant derivatives of R57 *SGO1 gdh1-2/2*, wild type for the indicated genes. Error bars represent plus or minus one standard deviation. The dashed line is a visual reference for the level achieved by the wildtype.
