# Supplementary material for: Determinants of selection in yeast evolved by genome shuffling
Source: Biotechnol Biofuels. 2018 Oct 16;11:282. doi: 10.1186/s13068-018-1283-9 (PMC6190656; doi:10.1186/s13068-018-1283-9)
Supplement: Supplementary file 1 — Additional file 1: Table S1. Population sequencing and alignment metrics. Table S2. Amplicon sequencing and alignment metrics for genotyping of R57 backcrossing isolates. Table S3. List of primers used for production of Ion Torrent sequencing libraries. [file 13068_2018_1283_MOESM1_ESM.docx]

**Table S1.** Population sequencing and alignment metrics

| **Pool** | **Number of reads** | **Read length** | **Mean base quality** | **Mean depth of coverage** | **Depth of coverage 1st percentile** | **Depth of coverage 99th percentile** | **Mean mapping quality** |
| --- | --- | --- | --- | --- | --- | --- | --- |
| **a** | 335377268 | 100 | 35.28 | 986 | 408 | 1827 | 47.23 |
| **α** | 488893080 | 100 | 35.09 | 1126 | 471 | 2172 | 44.46 |
| **R1** | 408135762 | 100 | 34.60 | 1182 | 460 | 2208 | 47.64 |
| **R2** | 311653312 | 100 | 35.15 | 712 | 295 | 1400 | 41.11 |
| **R3** | 388469930 | 100 | 34.73 | 807 | 328 | 1699 | 39.04 |
| **R4** | 382880430 | 100 | 34.46 | 1551 | 594 | 4008 | 48.58 |
| **R5** | 392463442 | 100 | 36.17 | 1270 | 549 | 2529 | 46.98 |
| **Average** | 386839032 | 100 | 35.07 | 1091 | 444 | 2263 | 45.01 |

**Table S2**. Amplicon sequencing and alignment metrics for genotyping of F2 backcrossing isolates

| **Locus** | **Average depth** | **Max depth** | **Min depth** | **Nb below 30** |
| --- | --- | --- | --- | --- |
| *ARO1-1283* | 1082 | 1801 | 1 | 1 |
| *ARO1-1284* | 259 | 736 | 0 | 1 |
| *ART5-454* | 1193 | 1826 | 76 | 0 |
| *BCS1-(-43)* | 126 | 214 | 5 | 5 |
| *DOP1-40* | 807 | 1588 | 0 | 1 |
| *FIT3-(-42)* | 229 | 657 | 79 | 0 |
| *GDH1-68* | 1149 | 1635 | 227 | 0 |
| *GDH1-47* | 1033 | 1488 | 181 | 0 |
| *GSH1-(-73)* | 497 | 875 | 5 | 3 |
| *MAL11-482* | 1546 | 2253 | 3 | 1 |
| *MAL11-310* | 711 | 1816 | 0 | 1 |
| *NOP58-25* | 506 | 772 | 1 | 2 |
| *NRG1-137* | 672 | 1436 | 39 | 0 |
| *PBP1-(-191)* | 258 | 606 | 0 | 2 |
| *SGO1-575* | 1087 | 1530 | 6 | 2 |
| *SSA1-91* | 890 | 1711 | 0 | 3 |
| *STE5_1512* | 268 | 541 | 2 | 1 |
| *STE5_3649* | 1090 | 1712 | 1 | 6 |
| *TOF2-2141* | 1238 | 1861 | 11 | 2 |
| *UBP7-2466* | 395 | 741 | 0 | 2 |
| *YNL058C-7* | 1590 | 3157 | 3 | 2 |
| *ALL* | 794 | 3157 | 0 | 35 |

**Table S3.** List of primers used for production of Ion Torrent sequencing library

| **Function** | **Name** | **Sequence (5' -> 3')** |
| --- | --- | --- |
| **locus specific primers** | itARO1_F | CGTTCAACCTTGTCCAACAGTGTAAGTGCTATGGTGACTCCGCTCA |
|  | itART5_F | CGTTCAACCTTGTCCAACAGTGACGGGCGTTTTCTCTTAAACCCTC |
|  | itBCS1_F | CGTTCAACCTTGTCCAACAGTGCGACATGGTAGATTGAGGGCACAC |
|  | itDOP1_F | CGTTCAACCTTGTCCAACAGTGCTCGCTCGACATTAGCACGAAACT |
|  | itFIT3_F | CGTTCAACCTTGTCCAACAGTGAGGAAGACCAACGAACTTGAAACAGT |
|  | itGDH1_F | CGTTCAACCTTGTCCAACAGTGACCCTTGTCATTTTCCCAGGTGAC |
|  | itGSH1_F | CGTTCAACCTTGTCCAACAGTGGTGCCCAAAGCTAAGAGTCCCATT |
|  | itMAL11_F | CGTTCAACCTTGTCCAACAGTGTGCAAACCAATCATCTCACCACAA |
|  | itNOP58_F | CGTTCAACCTTGTCCAACAGTGTCCAAATAAAAGGGAACGCGAGGG |
|  | itNRG1_F | CGTTCAACCTTGTCCAACAGTGGGCTCCCACTTTTCAGAGCCATTT |
|  | itPBP1_F | CGTTCAACCTTGTCCAACAGTGTGCTTCCCCTTCAAGATCAATTCGG |
|  | itSGO1_F | CGTTCAACCTTGTCCAACAGTGACGATGGTCCAGACCTTGAACCTA |
|  | itSSA1_F | CGTTCAACCTTGTCCAACAGTGAGCTTGATTCTTAGCAGCATCACCA |
|  | itSTE5-1_F | CGTTCAACCTTGTCCAACAGTGTATCCCGAGCAAATGAGCCTCCAA |
|  | itSTE5-2_1_F | CGTTCAACCTTGTCCAACAGTGTCAGTACAGCCAGCTCTGATTCCT |
|  | itTOF2_F | CGTTCAACCTTGTCCAACAGTGTCCACATCCAGCATTGGTTTTCCA |
|  | itUBP7_F | CGTTCAACCTTGTCCAACAGTGTCAAGGGCAAATGGAGAACAGCTT |
|  | itYNL054C_F | CGTTCAACCTTGTCCAACAGTGTGCTCCACTGGAAGTTGAGAAAGC |
|  | itARO1_R | GAAGCGATGACTCGAGCGTATTAGGGCAGCAAGAATTAAAGCACGA |
|  | itART5_R | GAAGCGATGACTCGAGCGTATTAGCAACCAAGATATGGACACCAGC |
|  | itBCS1_R | GAAGCGATGACTCGAGCGTATTTGAGTTACCCTTAAAGAGTGCCGA |
|  | itDOP1_R | GAAGCGATGACTCGAGCGTATTGCAGCTTTTTCTGAGAGGATCGACA |
|  | itFIT3_R | GAAGCGATGACTCGAGCGTATTCCCTTGCTGCCGTCATGTTATTGT |
|  | itGDH1_R | GAAGCGATGACTCGAGCGTATTTGTCAGAGCCAGAATTTCAACAAGC |
|  | itGSH1_R | GAAGCGATGACTCGAGCGTATTGTTGGATCTTTCCACGTGGTCTCG |
|  | itMAL11_R | GAAGCGATGACTCGAGCGTATTATGACTTTGAAGCAGGCGTTGCTA |
|  | itNOP58_R | GAAGCGATGACTCGAGCGTATTGCTAAGAAGGAAAAGAAGGACAAGAAGG |
|  | itNRG1_R | GAAGCGATGACTCGAGCGTATTCGGGAATCTCCGCATTTGATGGAA |
|  | itPBP1_R | GAAGCGATGACTCGAGCGTATTGAAGGAAAGCTCAATTGGGAGGCA |
|  | itSGO1_R | GAAGCGATGACTCGAGCGTATTCATGGCTTCGTTTTCACCGGTTTC |
|  | itSSA1_R | GAAGCGATGACTCGAGCGTATTTACTCGTGTGTTGCTCACTTTGCT |
|  | itSTE5-1_R | GAAGCGATGACTCGAGCGTATTCTTAAGTGGCCACATGCAAGCTCT |
|  | itSTE5-2_1_R | GAAGCGATGACTCGAGCGTATTTGAACTGCGTCTGGTTATGCCATC |
|  | itTOF2_R | GAAGCGATGACTCGAGCGTATTCCAGGTCGCATTCTTCACCTTCTT |
|  | itUBP7_R | GAAGCGATGACTCGAGCGTATTTCACCAGAGAGTACCTCATCGCTT |
|  | itYNL054C_R | GAAGCGATGACTCGAGCGTATTAGAAAGAAACATCGGCAGAGATGGT |
| **Adapter and barcode addition** | A_M1_F | CCATCTCATCCCTGCGTGTCTCCGACTCAGCATATGCGCGTTCAACCTTGTCCAACAGTG |
|  | A_M2_F | CCATCTCATCCCTGCGTGTCTCCGACTCAGTGATCGACCGTTCAACCTTGTCCAACAGTG |
|  | A_M3_F | CCATCTCATCCCTGCGTGTCTCCGACTCAGTCAGTCAGCGTTCAACCTTGTCCAACAGTG |
|  | A_M4_F | CCATCTCATCCCTGCGTGTCTCCGACTCAGTCAGTACGCGTTCAACCTTGTCCAACAGTG |
|  | A_M5_F | CCATCTCATCCCTGCGTGTCTCCGACTCAGGTCTACGACGTTCAACCTTGTCCAACAGTG |
|  | A_M6_F | CCATCTCATCCCTGCGTGTCTCCGACTCAGGACTGTCACGTTCAACCTTGTCCAACAGTG |
|  | A_M7_F | CCATCTCATCCCTGCGTGTCTCCGACTCAGTACGAGTCCGTTCAACCTTGTCCAACAGTG |
|  | A_M8_F | CCATCTCATCCCTGCGTGTCTCCGACTCAGAGTCGATCCGTTCAACCTTGTCCAACAGTG |
|  | A_M9_F | CCATCTCATCCCTGCGTGTCTCCGACTCAGACGTGTACCGTTCAACCTTGTCCAACAGTG |
|  | A_M10_F | CCATCTCATCCCTGCGTGTCTCCGACTCAGCAGCATGTCGTTCAACCTTGTCCAACAGTG |
|  | A_M11_F | CCATCTCATCCCTGCGTGTCTCCGACTCAGTCGACATGCGTTCAACCTTGTCCAACAGTG |
|  | A_M12_F | CCATCTCATCCCTGCGTGTCTCCGACTCAGCACTGATGCGTTCAACCTTGTCCAACAGTG |
|  | A_M13_F | CCATCTCATCCCTGCGTGTCTCCGACTCAGGTAGCACTCGTTCAACCTTGTCCAACAGTG |
|  | A_M14_F | CCATCTCATCCCTGCGTGTCTCCGACTCAGCTGAGCTACGTTCAACCTTGTCCAACAGTG |
|  | A_M15_F | CCATCTCATCCCTGCGTGTCTCCGACTCAGCATGCATGCGTTCAACCTTGTCCAACAGTG |
|  | A_M16_F | CCATCTCATCCCTGCGTGTCTCCGACTCAGCGATACTGCGTTCAACCTTGTCCAACAGTG |
|  | A_M17_F | CCATCTCATCCCTGCGTGTCTCCGACTCAGCATCAGTGCGTTCAACCTTGTCCAACAGTG |
|  | A_M18_F | CCATCTCATCCCTGCGTGTCTCCGACTCAGCTAGTACGCGTTCAACCTTGTCCAACAGTG |
|  | A_M19_F | CCATCTCATCCCTGCGTGTCTCCGACTCAGATGCTACGCGTTCAACCTTGTCCAACAGTG |
|  | A_M20_F | CCATCTCATCCCTGCGTGTCTCCGACTCAGGCATCGTACGTTCAACCTTGTCCAACAGTG |
|  | A_M21_F | CCATCTCATCCCTGCGTGTCTCCGACTCAGAGTCTCGACGTTCAACCTTGTCCAACAGTG |
|  | A_M22_F | CCATCTCATCCCTGCGTGTCTCCGACTCAGTGTCACGACGTTCAACCTTGTCCAACAGTG |
|  | A_M23_F | CCATCTCATCCCTGCGTGTCTCCGACTCAGCGTAGTCACGTTCAACCTTGTCCAACAGTG |
|  | A_M24_F | CCATCTCATCCCTGCGTGTCTCCGACTCAGCTGACTAGCGTTCAACCTTGTCCAACAGTG |
|  | A_M25_F | CCATCTCATCCCTGCGTGTCTCCGACTCAGGATCTACGCGTTCAACCTTGTCCAACAGTG |
|  | A_M26_F | CCATCTCATCCCTGCGTGTCTCCGACTCAGCTATGAGCCGTTCAACCTTGTCCAACAGTG |
|  | A_M27_F | CCATCTCATCCCTGCGTGTCTCCGACTCAGGCATGCTACGTTCAACCTTGTCCAACAGTG |
|  | A_M28_F | CCATCTCATCCCTGCGTGTCTCCGACTCAGTCGATCGACGTTCAACCTTGTCCAACAGTG |
|  | A_M29_F | CCATCTCATCCCTGCGTGTCTCCGACTCAGGCACTGATCGTTCAACCTTGTCCAACAGTG |
|  | A_M30_F | CCATCTCATCCCTGCGTGTCTCCGACTCAGCTGTACGACGTTCAACCTTGTCCAACAGTG |
|  | A_M31_F | CCATCTCATCCCTGCGTGTCTCCGACTCAGGCTAGCATCGTTCAACCTTGTCCAACAGTG |
|  | A_M32_F | CCATCTCATCCCTGCGTGTCTCCGACTCAGTAGACGCTCGTTCAACCTTGTCCAACAGTG |
|  | A_M33_F | CCATCTCATCCCTGCGTGTCTCCGACTCAGCGTACTGACGTTCAACCTTGTCCAACAGTG |
|  | A_M34_F | CCATCTCATCCCTGCGTGTCTCCGACTCAGGACTGCATCGTTCAACCTTGTCCAACAGTG |
|  | A_M35_F | CCATCTCATCCCTGCGTGTCTCCGACTCAGACTCGTGACGTTCAACCTTGTCCAACAGTG |
|  | A_M36_F | CCATCTCATCCCTGCGTGTCTCCGACTCAGACGTGCATCGTTCAACCTTGTCCAACAGTG |
|  | A_M37_F | CCATCTCATCCCTGCGTGTCTCCGACTCAGGTCATACGCGTTCAACCTTGTCCAACAGTG |
|  | A_M38_F | CCATCTCATCCCTGCGTGTCTCCGACTCAGACGTCGTACGTTCAACCTTGTCCAACAGTG |
|  | A_M39_F | CCATCTCATCCCTGCGTGTCTCCGACTCAGACAGTGTCCGTTCAACCTTGTCCAACAGTG |
|  | A_M40_F | CCATCTCATCCCTGCGTGTCTCCGACTCAGCAGTCATGCGTTCAACCTTGTCCAACAGTG |
|  | A_M41_F | CCATCTCATCCCTGCGTGTCTCCGACTCAGTCAGACGTCGTTCAACCTTGTCCAACAGTG |
|  | A_M42_F | CCATCTCATCCCTGCGTGTCTCCGACTCAGGACTCTGACGTTCAACCTTGTCCAACAGTG |
|  | A_M43_F | CCATCTCATCCCTGCGTGTCTCCGACTCAGATAGTCGCCGTTCAACCTTGTCCAACAGTG |
|  | A_M44_F | CCATCTCATCCCTGCGTGTCTCCGACTCAGTACACGTGCGTTCAACCTTGTCCAACAGTG |
|  | A_M45_F | CCATCTCATCCCTGCGTGTCTCCGACTCAGGTACTGACCGTTCAACCTTGTCCAACAGTG |
|  | A_M46_F | CCATCTCATCCCTGCGTGTCTCCGACTCAGACTGTAGCCGTTCAACCTTGTCCAACAGTG |
|  | A_M47_F | CCATCTCATCCCTGCGTGTCTCCGACTCAGGTCTGACACGTTCAACCTTGTCCAACAGTG |
|  | A_M48_F | CCATCTCATCCCTGCGTGTCTCCGACTCAGATGATCGCCGTTCAACCTTGTCCAACAGTG |
|  | A_M49_F | CCATCTCATCCCTGCGTGTCTCCGACTCAGAGCTGCATCGTTCAACCTTGTCCAACAGTG |
|  | A_M50_F | CCATCTCATCCCTGCGTGTCTCCGACTCAGTGACGTACCGTTCAACCTTGTCCAACAGTG |
|  | A_M51_F | CCATCTCATCCCTGCGTGTCTCCGACTCAGTACGTCAGCGTTCAACCTTGTCCAACAGTG |
|  | A_M52_F | CCATCTCATCCCTGCGTGTCTCCGACTCAGCGATCGTACGTTCAACCTTGTCCAACAGTG |
|  | A_M53_F | CCATCTCATCCCTGCGTGTCTCCGACTCAGCGATGCATCGTTCAACCTTGTCCAACAGTG |
|  | A_M54_F | CCATCTCATCCCTGCGTGTCTCCGACTCAGAGTCATGCCGTTCAACCTTGTCCAACAGTG |
|  | A_M55_F | CCATCTCATCCCTGCGTGTCTCCGACTCAGGTCAGCATCGTTCAACCTTGTCCAACAGTG |
|  | A_M56_F | CCATCTCATCCCTGCGTGTCTCCGACTCAGCGCATATGCGTTCAACCTTGTCCAACAGTG |
|  | A_M57_F | CCATCTCATCCCTGCGTGTCTCCGACTCAGACACGTGTCGTTCAACCTTGTCCAACAGTG |
|  | A_M58_F | CCATCTCATCCCTGCGTGTCTCCGACTCAGGTACGTACCGTTCAACCTTGTCCAACAGTG |
|  | A_M59_F | CCATCTCATCCCTGCGTGTCTCCGACTCAGCGTGACATCGTTCAACCTTGTCCAACAGTG |
|  | A_M60_F | CCATCTCATCCCTGCGTGTCTCCGACTCAGGCAGTCTACGTTCAACCTTGTCCAACAGTG |
|  | A_M61_F | CCATCTCATCCCTGCGTGTCTCCGACTCAGCGCTATGACGTTCAACCTTGTCCAACAGTG |
|  | A_M62_F | CCATCTCATCCCTGCGTGTCTCCGACTCAGGTACAGCTCGTTCAACCTTGTCCAACAGTG |
|  | A_M63_F | CCATCTCATCCCTGCGTGTCTCCGACTCAGTAGCACGTCGTTCAACCTTGTCCAACAGTG |
|  | A_M64_F | CCATCTCATCCCTGCGTGTCTCCGACTCAGTCTGAGCACGTTCAACCTTGTCCAACAGTG |
|  | A_M65_F | CCATCTCATCCCTGCGTGTCTCCGACTCAGTACATGCGCGTTCAACCTTGTCCAACAGTG |
|  | A_M66_F | CCATCTCATCCCTGCGTGTCTCCGACTCAGAGCTACGTCGTTCAACCTTGTCCAACAGTG |
|  | A_M67_F | CCATCTCATCCCTGCGTGTCTCCGACTCAGTACTGAGCCGTTCAACCTTGTCCAACAGTG |
|  | A_M68_F | CCATCTCATCCCTGCGTGTCTCCGACTCAGTACGAGCTCGTTCAACCTTGTCCAACAGTG |
|  | A_M69_F | CCATCTCATCCCTGCGTGTCTCCGACTCAGAGTACTCGCGTTCAACCTTGTCCAACAGTG |
|  | A_M70_F | CCATCTCATCCCTGCGTGTCTCCGACTCAGAGATCTGCCGTTCAACCTTGTCCAACAGTG |
|  | A_M71_F | CCATCTCATCCCTGCGTGTCTCCGACTCAGATGTCAGCCGTTCAACCTTGTCCAACAGTG |
|  | A_M72_F | CCATCTCATCCCTGCGTGTCTCCGACTCAGCTAGTGACCGTTCAACCTTGTCCAACAGTG |
|  | A_M73_F | CCATCTCATCCCTGCGTGTCTCCGACTCAGTGCATAGCCGTTCAACCTTGTCCAACAGTG |
|  | A_M74_F | CCATCTCATCCCTGCGTGTCTCCGACTCAGATCGCTAGCGTTCAACCTTGTCCAACAGTG |
|  | A_M75_F | CCATCTCATCCCTGCGTGTCTCCGACTCAGGCTCTAGACGTTCAACCTTGTCCAACAGTG |
|  | A_M76_F | CCATCTCATCCCTGCGTGTCTCCGACTCAGGCGATCATCGTTCAACCTTGTCCAACAGTG |
|  | A_M77_F | CCATCTCATCCCTGCGTGTCTCCGACTCAGTACGTACGCGTTCAACCTTGTCCAACAGTG |
|  | A_M78_F | CCATCTCATCCCTGCGTGTCTCCGACTCAGTGCAGTACCGTTCAACCTTGTCCAACAGTG |
|  | A_M79_F | CCATCTCATCCCTGCGTGTCTCCGACTCAGTGAGTCACCGTTCAACCTTGTCCAACAGTG |
|  | A_M80_F | CCATCTCATCCCTGCGTGTCTCCGACTCAGACTGTCGACGTTCAACCTTGTCCAACAGTG |
|  | A_M81_F | CCATCTCATCCCTGCGTGTCTCCGACTCAGCGAGTCTACGTTCAACCTTGTCCAACAGTG |
|  | A_M82_F | CCATCTCATCCCTGCGTGTCTCCGACTCAGTCGTAGACCGTTCAACCTTGTCCAACAGTG |
|  | A_M83_F | CCATCTCATCCCTGCGTGTCTCCGACTCAGCGTAGCATCGTTCAACCTTGTCCAACAGTG |
|  | A_M84_F | CCATCTCATCCCTGCGTGTCTCCGACTCAGACTGTGCACGTTCAACCTTGTCCAACAGTG |
|  | A_M85_F | CCATCTCATCCCTGCGTGTCTCCGACTCAGTAGCTACGCGTTCAACCTTGTCCAACAGTG |
|  | A_M86_F | CCATCTCATCCCTGCGTGTCTCCGACTCAGCATCGAGTCGTTCAACCTTGTCCAACAGTG |
|  | A_M87_F | CCATCTCATCCCTGCGTGTCTCCGACTCAGATACGTCGCGTTCAACCTTGTCCAACAGTG |
|  | A_M88_F | CCATCTCATCCCTGCGTGTCTCCGACTCAGCGATGTCACGTTCAACCTTGTCCAACAGTG |
|  | A_M89_F | CCATCTCATCCCTGCGTGTCTCCGACTCAGTAGTCAGCCGTTCAACCTTGTCCAACAGTG |
|  | A_M90_F | CCATCTCATCCCTGCGTGTCTCCGACTCAGATCAGCGTCGTTCAACCTTGTCCAACAGTG |
|  | A_M91_F | CCATCTCATCCCTGCGTGTCTCCGACTCAGCAGTGACTCGTTCAACCTTGTCCAACAGTG |
|  | A_M92_F | CCATCTCATCCCTGCGTGTCTCCGACTCAGATGCTGCACGTTCAACCTTGTCCAACAGTG |
|  | A_M93_F | CCATCTCATCCCTGCGTGTCTCCGACTCAGTGATCACGCGTTCAACCTTGTCCAACAGTG |
|  | A_M94_F | CCATCTCATCCCTGCGTGTCTCCGACTCAGTACGCGTACGTTCAACCTTGTCCAACAGTG |
|  | A_M95_F | CCATCTCATCCCTGCGTGTCTCCGACTCAGGTGTACACCGTTCAACCTTGTCCAACAGTG |
|  | A_M96_F | CCATCTCATCCCTGCGTGTCTCCGACTCAGTAGCGTCACGTTCAACCTTGTCCAACAGTG |
|  | P1_M_R | CCTCTCTATGGGCAGTCGGTGATGAAGCGATGACTCGAGCGTATT |
