# Supplementary figures and images for: Determinants of selection in yeast evolved by genome shuffling
Source: Biotechnol Biofuels. 2018 Oct 16;11:282. doi: 10.1186/s13068-018-1283-9 (PMC6190656; doi:10.1186/s13068-018-1283-9)

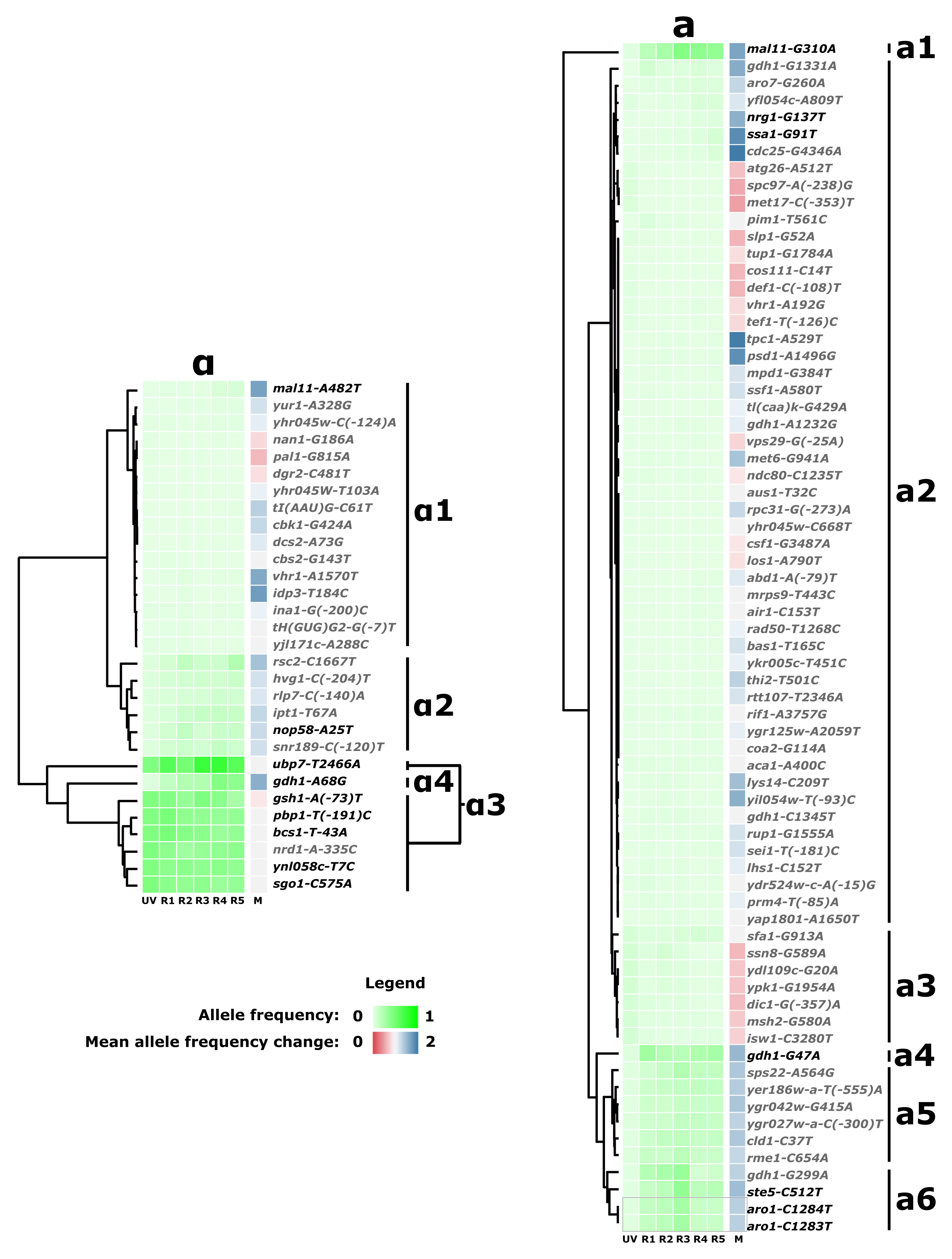

Supplement: Supplementary file 3 — Additional file 3: Figure S1. Evolutionary trajectories for all non-silent mutations identified by population genome sequencing at 6 time points. Mutations arose either in the MATα (left) or MATa (right) haploid populations. On the vertical axis are the names of the mutations, giving the closest gene, coordinates with respect to that gene and the nature of the nucleotide substitution. On the horizontal axis are each of the six evolutionary time points (UV, R1, R2, R3, R4, R5) and the mean allele frequency change (M). Frequencies of the mutant alleles are represented by shades of green. Mean allele frequency changes are represented in shades of red (M < 1, declining frequency) or blue (M > 1, increasing frequency). Hierarchical clustering of individual evolutionary trajectories is represented by dendrograms on the left. Mutations were assigned to groups of mutations (a1-5, α1-4) on the basis of this clustering. Mutations present in highly tolerant mutant R57 are highlighted in bold. [file 13068_2018_1283_MOESM3_ESM.png]

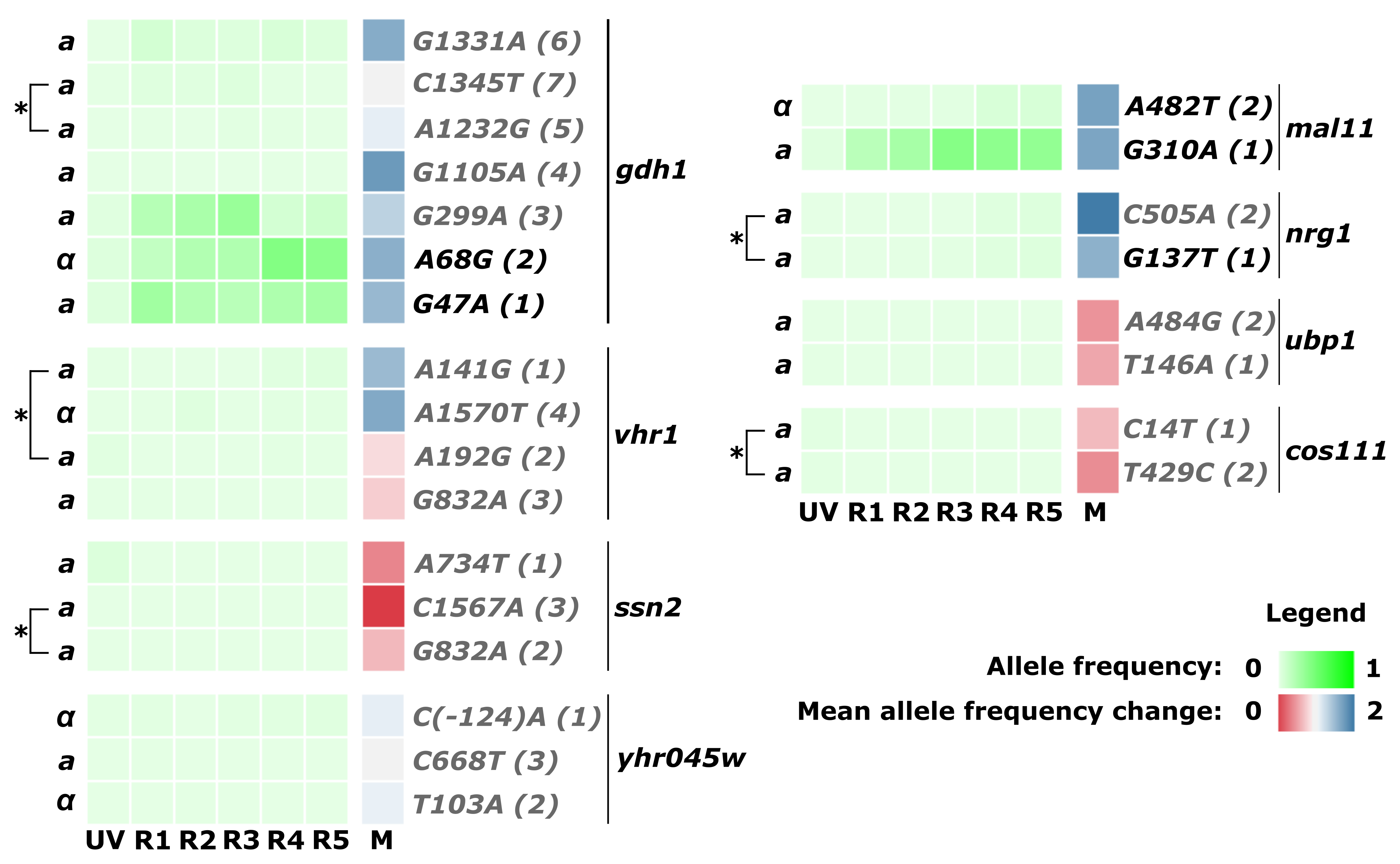

Supplement: Supplementary file 4 — Additional file 4: Figure S2. Evolutionary trajectories and apparent selection of all mutation hotspots identified by population sequencing. Mutations arose either in the MATα (left) or MATa (right) as indicated immediately to the left of each mutation. On the vertical axis are the names of the mutations, giving the closest gene, coordinates with respect to that gene and the nature of the nucleotide substitution. On the horizontal axis are each of the six evolutionary time points (UV, R1, R2, R3, R4, R5) and the mean allele frequency change (M). Frequencies of the mutant alleles are represented by shades of green. Mean allele frequency changes are represented in shades of red (M < 1, declining frequency) or blue (M > 1, increasing frequency). Mutations linked by connectors and marked with an asterisk indicate pairs with significantly similar initial frequency (binomial test, p > 0.05). [file 13068_2018_1283_MOESM4_ESM.png]

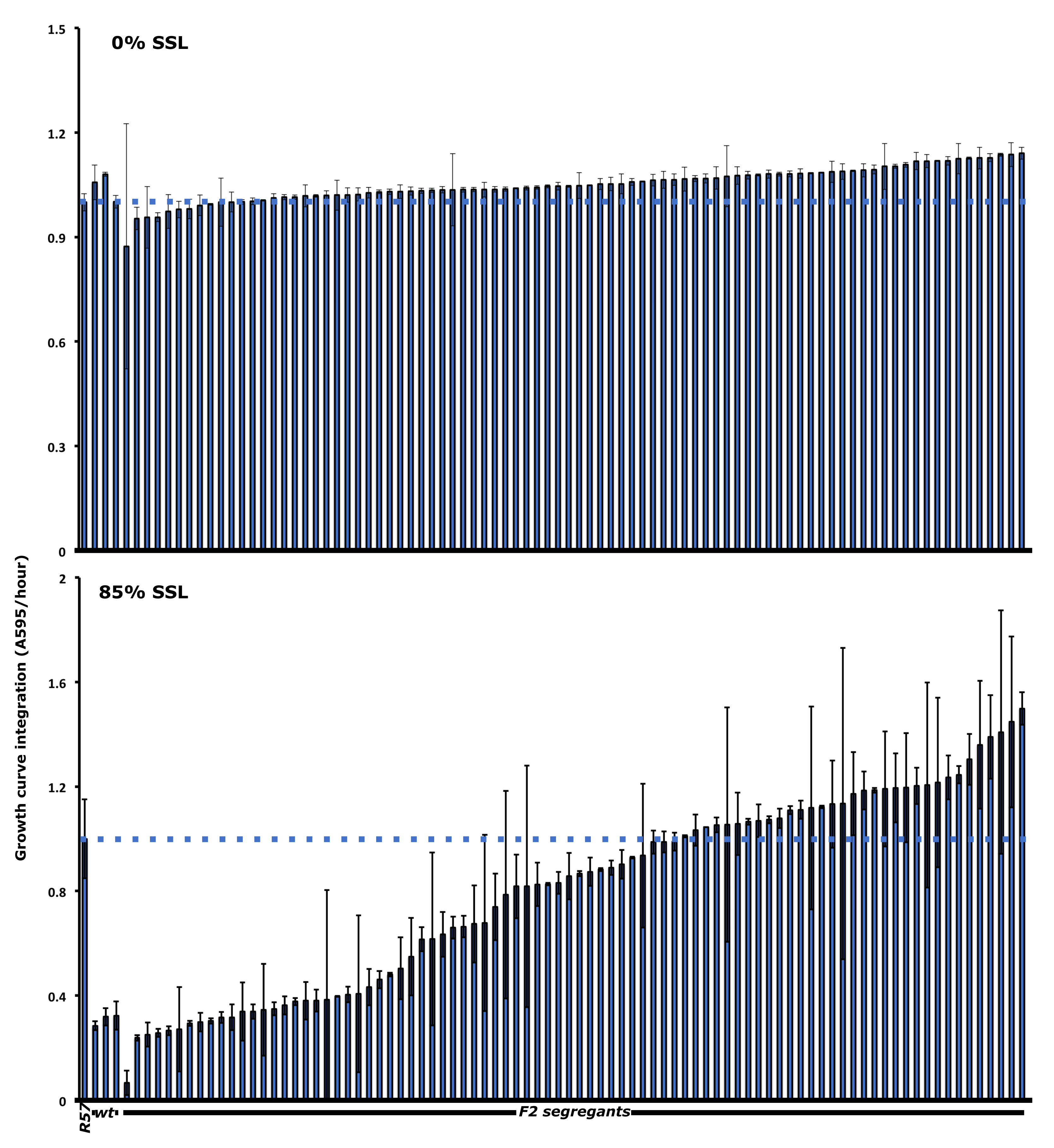

Supplement: Supplementary file 6 — Additional file 6: Figure S3. Backcrossing of R57 with wild type cells generates strains presenting a wide spectrum of fitness in SSL. Growth in the presence and absence of SSL is reported for R57, various wild type cell types and 86 F2 isolates from backcrossing of R57 and CEN.PK113-1A. Error bars represent plus or minus one standard deviation. The dashed line is a visual reference for the level achieved by the wildtype. [file 13068_2018_1283_MOESM6_ESM.png]

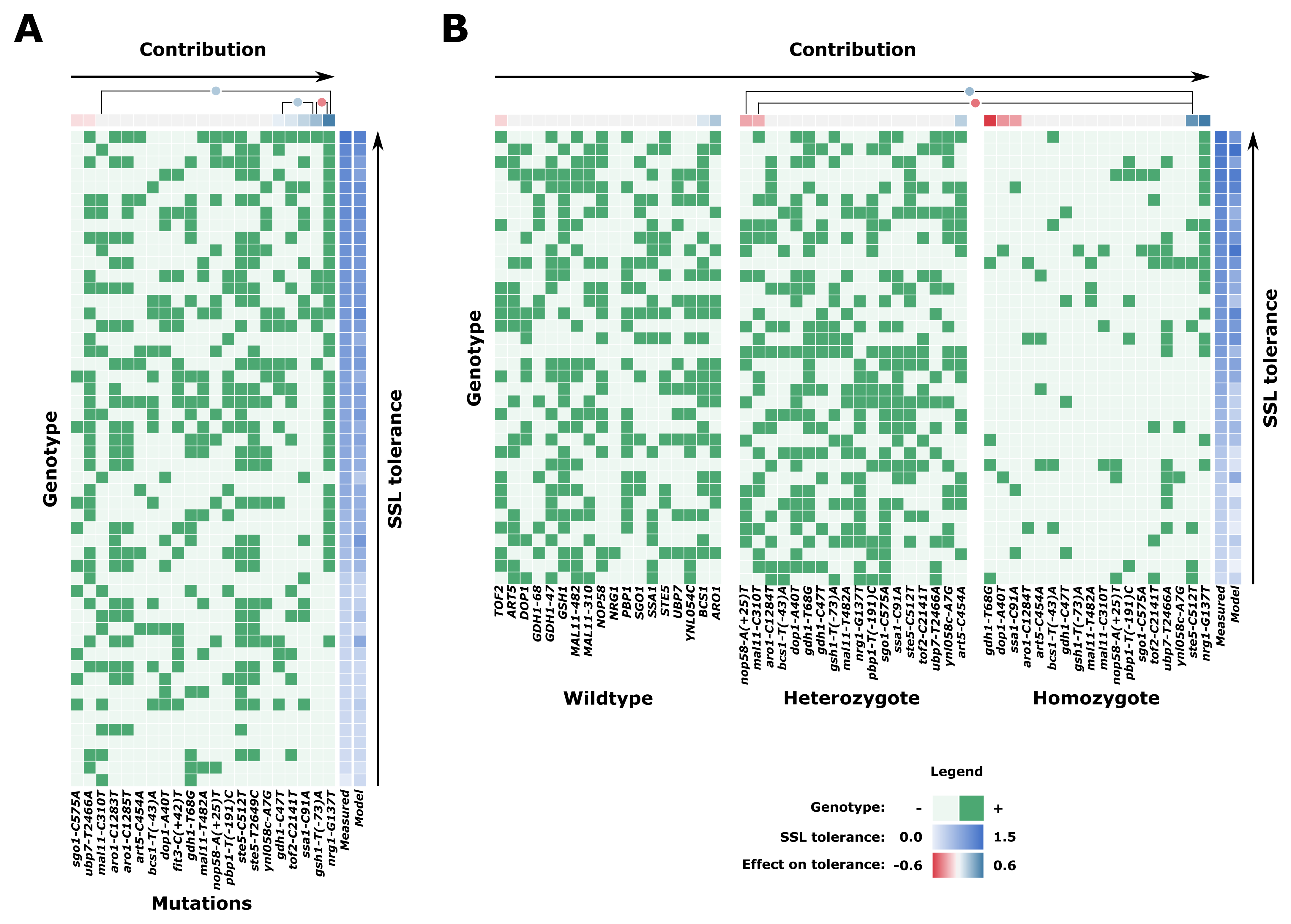

Supplement: Supplementary file 7 — Additional file 7: Figure S4. Genotyping of second generation segregants from backcrossing of R57 and wild type yeast suggest a model of SNP contributions to the SSL tolerance phenotype. Haploid (A) and diploid (B) isolates are scored in green for the genotypes indicated at the bottom. Growth in 85% SSL is scored in shades of blue on the right. Each row represents a single strain. Contribution to the phenotype of the indicated genotypes was inferred by multiple linear regression, yielding coefficients represented at the top in shades of red (diminishes fitness) to blue (increases fitness). Modeling of genetic interactions was attempted and the resulting coefficients are represented as circles at the top of the heatmaps. Growth in SSL predicted by the linear model is reported in shades of blue in the rightmost column, showing the level of agreement between the model and the data. [file 13068_2018_1283_MOESM7_ESM.png]

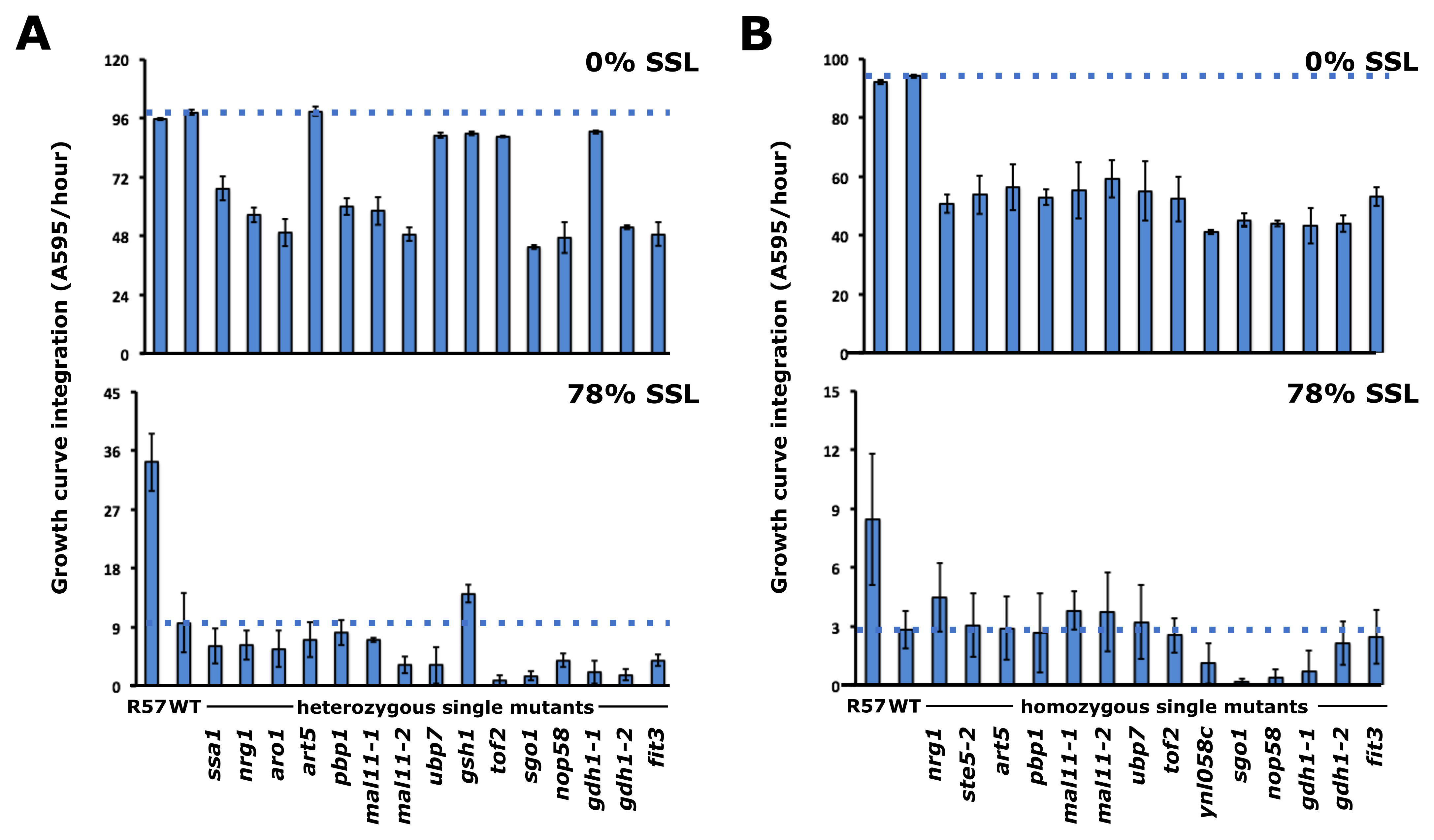

Supplement: Supplementary file 8 — Additional file 8: Figure S5. Single mutations are not sufficient to detect an increase in SSL tolerance in diploid cells. Area under the growth curve in the presence and absence of SSL for heterozygous (A) and homozygous (B) single diploid mutants is reported. Error bars represent plus or minus one standard deviation. The dashed line is a visual reference for the level achieved by the wildtype. [file 13068_2018_1283_MOESM8_ESM.png]

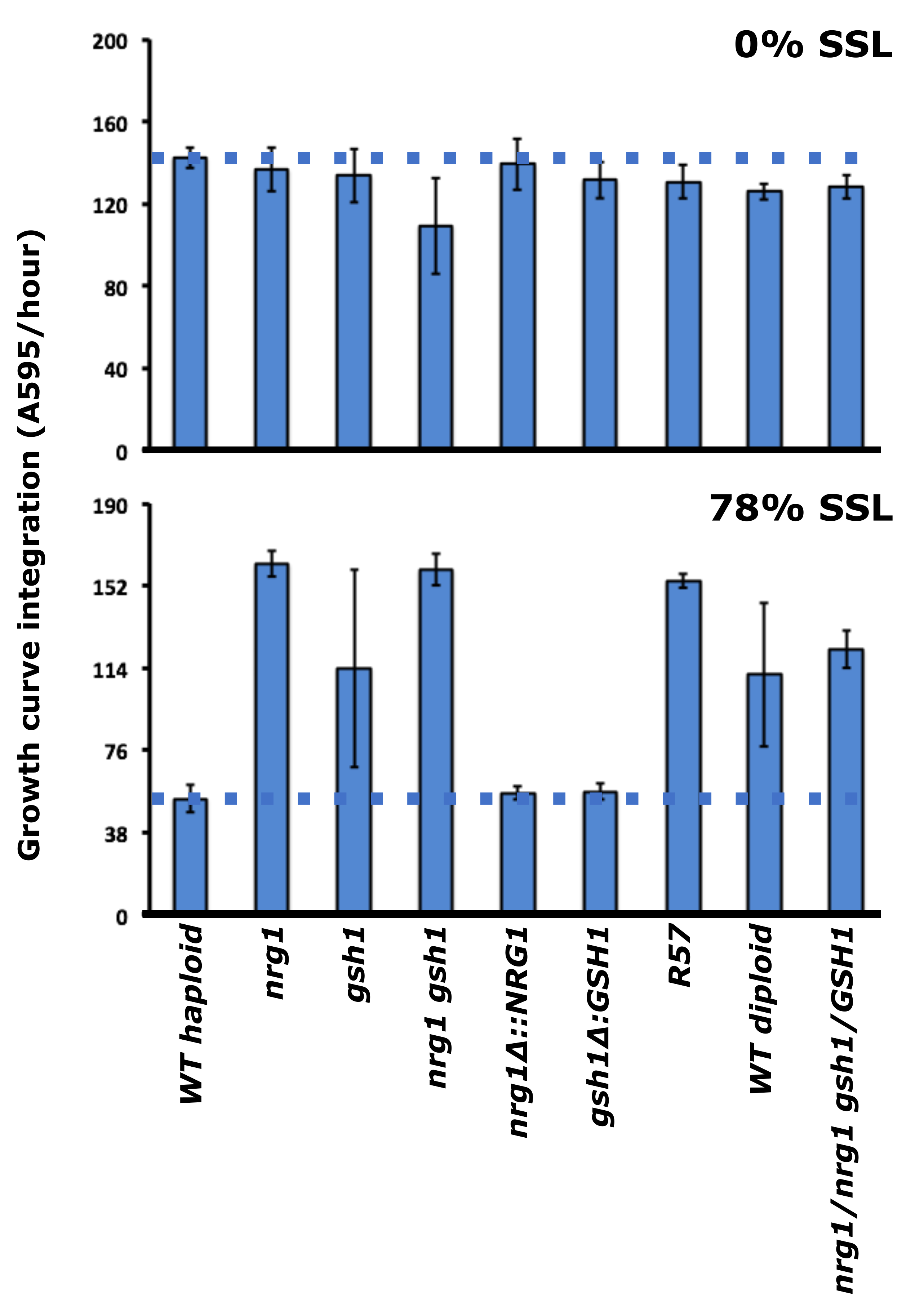

Supplement: Supplementary file 9 — Additional file 9: Figure S6. Reversion of nrg1 and gsh1 mutations leads to loss of the SSL tolerance phenotype in haploid single mutants. Area under the growth curve for nrg1 and gsh1 double mutants, haploid (nrg1 gsh1) and diploid (nrg1/nrg1 gsh1/GSH1) is also reported. Error bars represent plus or minus one standard deviation. The dashed line is a visual reference for the level achieved by the wildtype. [file 13068_2018_1283_MOESM9_ESM.png]

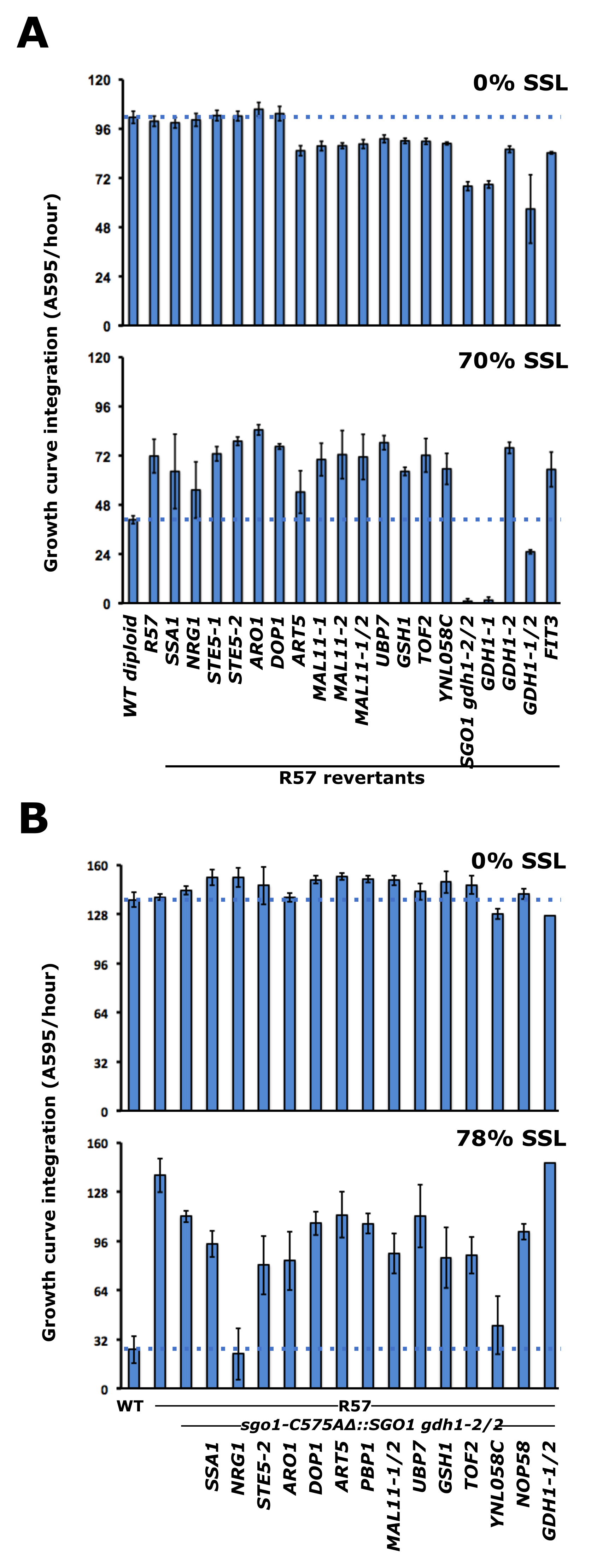

Supplement: Supplementary file 10 — Additional file 10: Figure S7. Growth in the presence and absence of SSL of single and double revertants identifies mutations contributing to the SSL tolerance phenotype. Area under the growth curve in the presence and absence of SSL is reported for (A) single haploid mutants, (B) single revertant derivatives of R57 and (C) revertant derivatives of R57 SGO1 gdh1-2/2, wild type for the indicated genes. Error bars represent plus or minus one standard deviation. The dashed line is a visual reference for the level achieved by the wildtype. [file 13068_2018_1283_MOESM10_ESM.png]
