# Supplementary material for: Determinants of selection in yeast evolved by genome shuffling
Source: Biotechnol Biofuels. 2018 Oct 16;11:282. doi: 10.1186/s13068-018-1283-9 (PMC6190656; doi:10.1186/s13068-018-1283-9)
Supplement: Supplementary file 5 — Additional file 5: Supporting methods. Detailed methods are provided for our SNP calling methodology, structural study of Gdh1p, determination of mating type and ploidy by PCR, and modeling of SSL tolerance by multiple linear regression. [file 13068_2018_1283_MOESM5_ESM.docx]

**Additional methods**

**Base error model, SNP calling and filtering**

Read counts for each genomic position were obtained from the indel-realigned output using bam-readcount [1] with minimum mapping quality and minimum base quality both set at 30. A base error model was computed from read counts using a methodology inspired from Barrick & Lenski [2]. Iterating over every nucleotide position in the reference genome, the probability of reading a base call given its quality score and the reference base was computed, generating a series of 4x4 matrices. Only positions with depth of coverage comprised between the 1^st^ and 99^th^ percentile were considered, to avoid regions of low coverage and because unusually high coverage was taken as an indication of incorrect alignment. The error model was then refined to correct for regions of the genome with above average proportion of mismatches with the reference. To this end, the reference genome was divided into 2000 nucleotide regions and local reference mismatch rates were compared to the genome-wide rate. For regions with an above average mismatch rate, the local mismatch rate was divided by the genome-wide mismatch rate to yield a correction factor, which was used to multiply mismatch probabilities predicted by the base error model. Uncorrected probabilities were used for regions with average and below average mismatch rates.

Using the same criteria as for error model construction, we iterated through every position in the reference genome, extracted base counts and compared them to error model predictions. Probability of the observed read counts given the error model was calculated using either a multinomial test (if depth of coverage < 170) or G-test (depth > 170). This probability was multiplied by the number of nucleotide positions in the reference genome to yield the expected number of positions with the same read counts found by chance in the dataset. If this number was less than one, a SNP was called. The list of SNP calls was compared to our previous re-sequencing of CEN.PK113-7D [3] to filter parental mutations and potential systematic errors. The pileup was extracted using samtools mpileup [4] for each mutant position in the resulting list of SNP calls, with a minimum quality threshold of 30 for both base reads and alignment. From the pileup, we filtered SNP calls for quality score bias. SNP calls for which the mean quality score of mismatch reads was lower than that of reference matching reads were submitted to a Kolmogorov-Smirnov test comparing the quality score distributions of match and mismatch reads. Significantly (p < 0.05) different quality score distributions led to SNP call rejection. SNP calls were further filtered for strand bias. Fisher's exact test was applied to determine if match and mismatch reads were similarly distributed between the forward and reverse strands. SNP calls for which mismatch reads showed a significant (p-value < 0.05) strand bias were filtered out. We next reasoned that mutations were unlikely to arise independently and be encountered simultaneously in both haploid mutant populations. We tested whether SNP calls encountered in both populations (Fisher's exact test, p < 0.05) had the same proportion of mismatches. If they did, a systematic error was suspected and further testing was performed. Mismatch count was compared between each haploid mutant population and the round 1 to 5 data. Fisher's method was used to combine the probabilities of the 10 tests. Highly significantly different (p < 0.001) proportions of mismatches between the 7 datasets led to SNPs being conserved. Otherwise, they were discarded. Filtering was completed by visual inspection of the alignments, using integrative genomics viewer [5,6]. High number of mismatches or secondary reads mapping in the vicinity of a SNP call led to its rejection. Only non-silent mutations with non-zero frequency at all six sampled time points were considered for all downstream analyses. Indel calling was attempted from pileup applying an error model and filtering as described for SNPs. We did not detect indels using this strategy.

**Multiple sequence alignment, homology modeling of *S. cerevisiae* Gdh1p and protein structure analysis**

Multiple sequence alignment of Gdh1p homologs was performed with Clustal Omega [7–9]. Homology modeling was performed automatically on the ModWeb server [10]. The best scoring model was based on the structure of *P. falciparum* glutamate dehydrogenase (52% identity, PDB ID: 2BMA, [11]). Exploration of the structure and mapping of Gdh1p substitutions was done with Pymol [12].

**Determination of mating type and ploidy by PCR**

For each strain, two PCR reactions were performed. A common *MAT* locus reverse primer (5’ AGTCACATCAAGATCGTTTATGG 3’) was used with a forward primer specific for either the *MATα* (5’ GCACGGAATATGGGACTACTTCG 3’) or *MATa* (5’ ACTCCACTTCAAGTAAGAGTTTG 3’) genes. Composition of the PCR reactions were as follows: 1 μl gDNA template, 0.5 μM each primer, 200 mM dNTPs, 1X KCl Taq buffer, 1.25 U Taq DNA polymerase in a 50 μl reaction volume. The cycling conditions were 95^o^C for 3 min, then 35 cycles of 95^o^C for 30s, 55^o^C for 30 s, 72^o^C for 1 min, followed by a 10 min incubation at 72^o^C and a final hold at 10^o^C. Detection of an approximately 500 bp bands by 0.8% agarose gel electrophoresis in both reactions indicated a diploid strain, while haploidy was inferred if only one reaction yielded a band. Mating type of haploids was deduced from the identity of the positive reaction (*MATα* or *MATa*).

**Multiple linear regression model of SSL tolerance in R57 backcrossed mutants**

Distinct linear models were computed from the haploid and diploid mutant datasets, using distinct methodologies, following guidelines from [13]. The full haploid dataset consisted of 52 data points, each corresponding to a single strain, including the wildtype CEN.PK113-1A strain. The diploid dataset was smaller, with 36 data points including strain R57. The general equation for both linear models is the of the form:

$$y=\beta_{0}+ \beta_{1}x_{1}+ \beta_{2}x_{2}+\ldots+\beta_{i}x_{i}+\ldots+ \beta_{k}x_{k}$$

where *y* represents the area under the growth curve in 85% SSL for a given strain, each *x_i_* represents the genotype of the strain at locus *i* of *k* mutant loci of interest, and each *β_i_* the linear coefficient associated with locus *i*, corresponding to its contribution to the SSL tolerance phenotype. Term *β_0_* corresponds to the value of *y* when all *x*_i_=0 which in haploids is the area under the growth curve in 85% SSL for wildtype cells. A straightforward biological interpretation for *β_0_* in diploids cannot be given.

Correlation (R^2^ >> 0.5) was observed in both datasets between pairs of mutations located at close coordinates on the same chromosomes (data not shown). Those were the *aro1-C1283T* and *aro1-C1284T*, *ste5-C512T* and *ste5-T2649C*, and *gdh1-C47T* and *fit3-C(+43)T* mutations. To avoid issues with collinearity, *aro1-C1283T*, *ste5-T2649C*  (silent) and *fit3-C(+43)T* (hypothesized to be inconsequential because not detected by population sequencing) were removed from our regression analyses. We also saw correlation between the *aro1* and *ste5* mutations, but decided to keep one representative of both, seeking identification of the best predictor of SSL tolerance by regression. Other correlations were measured, especially in the diploid dataset. Unlike the mutations mentioned above, we did not have biological reasons to exclude these variables from our analyses, and hypothesized that these correlations were coincidental.

For haploids, each mutant position has two possible genotypes, such that x_i_=0 for wildtype and x_i_=1 for mutant, and each *β_i_* is a direct estimate of the effect of mutation *i* on the phenotype. Our dataset thus included 18 potential explanatory variables. Using the *curve_fit* wrapper from the *Scipy* Python library, we estimated the linear coefficients for all combinations of *k*=1 to *k*=18 explanatory variables. For each of the resulting linear models, we computed *R^2^*, variance of the residuals, Mallow’s *C_p_* statistic, and p-values that each *β_i_=*0. For each value of *k* variables, we chose the model that minimized *C_p_*, then plotted this minimum *C_p_* against *k*. We found that *k*=8 explanatory variables minimized both *C_p_* and variance of residuals, and corresponded to the point where *R^2^* reached a plateau. Those three observations suggested that fitting with the selected eight variables maximized predictive power of the model while minimizing overfitting. P-values for the linear coefficients were all less than to 0.01.

For diploids, a single binary variable cannot be applied to each locus, because three possible genotypes are possible (wildtype, heterozygous mutant and homozygous mutant). Potential cases of heterozygote superiority or inferiority mean that a linear relationship between the phenotype and the number of mutant alleles at each locus cannot be assumed. Instead, three binary variables with associated linear coefficients are assigned to each locus for each potential genotype. The linear model is modified such that the *β_i_x_i_* become *β_iwt_x_iwt_*+ *β_ihet_x_ihet_*+ *β_ihom_x_ihom_.* For example, a heterozygous mutant would have x_iwt_=0, x_ihet_=1 and x_ihom_=0. This procedure multiplies the number of variables by three. This also means that the thorough approach used with the haploid dataset could not be applied to diploids. Indeed, the number of available data points (36) is smaller than the number of potential explanatory variables (54). We considered that regression with all potential explanatory variables was not possible without serious risks of overfitting. Without additional and biologically motivated constraints to impose onto the data, we chose not to attempt model fitting in those conditions. The consequence is that we could not compute the C_p_ statistic for diploid models. However, in haploid models, we noticed a strong correlation between the C_p_ statistic and the mean residual sum of square (MRSS) computed when performing leave-one-out cross validation (data not shown). We chose to use MRSS as a proxy for *C_p_* in diploid models. Further, the systematic fitting of linear models for all combinations of *k* variables among 54 potential explanatory variables rapidly becomes impractical for relatively low values of *k*. We chose to proceed in a stepwise manner. We started by identifying the model with *k*=1 that minimized MRSS. We then sampled all models with *k*=2 that included the variable chosen in the first step, and chose the one that led to the greatest reduction in MRSS. We incrementally added variables to the model in the same way until *k*=35. At each step, we monitored p-values for each β_i_. If any variable had a p-value > 0.01, we rejected it and computed all alternative models where that single variable was replaced, and selected the one that minimized MRSS. We plotted MRSS against *k* and selected the model that minimized MRSS.

We used an *ad hoc* methodology to consider potential interactions between pairs of mutations, and proceeded in the same way for haploids and diploids. We only considered pairs of variables already included in the final interaction-free models. Each pair of variables *i* and *j* was taken into account by adding a term to the linear equation of the form *β_ij_x_i_x_j_*. Interaction models were built using the same stepwise methodology followed for the diploid models. Thus, interaction terms were added incrementally to the existing, interaction-free model, choosing the one out of all possible pairs that led to the greatest reduction in MRSS. Addition of interaction terms was stopped when MRSS either reached a plateau or started increasing, indicating overfitting.

The selected linear models were validated using a variety of methods. Residuals were plotted against each of the model variables and against the measured values of *y* to detect fitting bias. We further tested normality of the distribution of residuals using a Kolmogorov-Smirnov test. For each data point (i.e. each strain) we computed Cook’s distance to identify outliers or influential points. We did detect an outlier in each model, but in the absence of strong biological reasons or other observations to reject them, we chose to keep those points as part of the natural variability of our data.

1. Larson D. Bam-readcount: program to generate metrics at single nucleotide positions from BAM files [Internet]. 2016. Available from: https://github.com/genome/bam-readcount

2. Barrick JE, Lenski RE. Genome-wide Mutational Diversity in an Evolving Population of Escherichia coli. Cold Spring Harb Symp Quant Biol. 2009;74:119–29.

3. Pinel D, Colatriano D, Jiang H, Lee H, Martin VJ. Deconstructing the genetic basis of spent sulphite liquor tolerance using deep sequencing of genome-shuffled yeast. Biotechnol Biofuels. 2015;8:53.

4. Li H, Handsaker B, Wysoker A, Fennell T, Ruan J, Homer N, et al. The Sequence Alignment/Map format and SAMtools. Bioinformatics. 2009;25:2078–9.

5. Robinson JT, Thorvaldsdóttir H, Winckler W, Guttman M, Lander ES, Getz G, et al. Integrative genomics viewer. Nat Biotechnol [Internet]. 2011;29:24–6. Available from: http://www.nature.com/nbt/journal/v29/n1/abs/nbt.1754.html%5Cnhttp://www.nature.com/nbt/journal/v29/n1/pdf/nbt.1754.pdf

6. Thorvaldsdóttir H, Robinson JT, Mesirov JP. Integrative Genomics Viewer (IGV): High-performance genomics data visualization and exploration. Brief Bioinform. 2013;14:178–92.

7. Sievers F, Wilm A, Dineen D, Gibson TJ, Karplus K, Li W, et al. Fast, scalable generation of high-quality protein multiple sequence alignments using Clustal Omega. Mol Syst Biol [Internet]. 2011;7:539. Available from: http://msb.embopress.org/content/7/1/539.abstract

8. Goujon M, McWilliam H, Li W, Valentin F, Squizzato S, Paern J, et al. A new bioinformatics analysis tools framework at EMBL-EBI. Nucleic Acids Res. 2010;38.

9. McWilliam H, Li W, Uludag M, Squizzato S, Park YM, Buso N, et al. Analysis Tool Web Services from the EMBL-EBI. Nucleic Acids Res. 2013;41.

10. Eswar N. Tools for comparative protein structure modeling and analysis. Nucleic Acids Res [Internet]. 2003;31:3375–80. Available from: http://www.nar.oupjournals.org/cgi/doi/10.1093/nar/gkg543%5Cnpapers2://publication/doi/10.1093/nar/gkg543

11. Werner C, Stubbs MT, Krauth-Siegel RL, Klebe G. The crystal structure of Plasmodium falciparum glutamate dehydrogenase, a putative target for novel antimalarial drugs. J Mol Biol. 2005;349:597–607.

12. Schrödinger LLC. The PyMOL Molecular Graphics System, Version 1.8. 2016.

13. Matthews DE. Multiple Linear Regression. 2nd editio. Encycl. Biostat. John Wiley & sons; 2005. p. 3428–41.
